# Novel Coconut Vinegar Attenuates Hepatic and Vascular Oxidative Stress in Rats Fed a High-Cholesterol Diet

**DOI:** 10.3389/fnut.2022.835278

**Published:** 2022-03-09

**Authors:** Wachirawadee Malakul, Porrnthanate Seenak, Noppadon Jumroon, Siwaret Arikit, Sarawut Kumphune, Nitirut Nernpermpisooth

**Affiliations:** ^1^Department of Physiology, Faculty of Medical Sciences, Naresuan University, Phitsanulok, Thailand; ^2^Integrative Biomedical Research Unit (IBRU), Faculty of Allied Health Sciences, Naresuan University, Phitsanulok, Thailand; ^3^Department of Cardio-Thoracic Technology, Faculty of Allied Health Sciences, Naresuan University, Phitsanulok, Thailand; ^4^Department of Medical Technology, Faculty of Allied Health Sciences, Naresuan University, Phitsanulok, Thailand; ^5^Department of Agronomy, Faculty of Agriculture at Kamphaeng Saen, Kasetsart University, Kamphaeng Saen Campus, Nakhon Pathom, Thailand; ^6^Biomedical Engineering Institute (BMEI), Chiang Mai University, Chiang Mai, Thailand

**Keywords:** coconut vinegar, dyslipidemia, hepatic oxidative stress, vascular oxidative stress, lipid peroxidation

## Abstract

**Background:**

Hypercholesterolemia is an independent modifiable risk factor that accelerates the development of both non-alcoholic fatty liver and atherosclerosis. Coconut water contains a variety of phytochemicals that make it appealing for producing vinegar. Coconut vinegar is rapidly gaining popularity for health benefits in Southeast Asia. The purpose of this study is to evaluate the effect of daily supplementation of coconut vinegar on hepatic and vascular oxidative stress in rats fed a high-cholesterol diet (HCD).

**Methods:**

Mature coconut water was fermented with coconut sap sugar using *Saccharomyces cerevisiae* and *Acetobacter aceti vat Europeans*, respectively. Bioactive compounds and antioxidant capacity of coconut vinegar were examined *in vitro*. Adult male Sprague–Dawley rats were randomly divided into four groups; the control group fed a standard diet (S), a group that received HCD (SC), a group that received HCD supplemented with coconut vinegar at a dose of 1 mL/kg/day (SCV), and a group that received HCD with atorvastatin at a dose of 30 mg/kg/day (SCA). After 8 weeks, serum metabolic profiles, fatty liver, hepatic, and vascular oxidative stress were determined.

**Results:**

In *in vitro* studies, coconut vinegar was rich in phenolic compounds and organic acids. The antioxidant capacity of 30 μL of coconut vinegar was 181.55 ± 8.15 μM Trolox equivalent antioxidant capacity (TEAC). In the HCD fed rats, daily supplementation of coconut vinegar reduced weight gain, serum triglycerides, and fasting blood sugar levels without renal or liver toxicity. In the liver, coconut vinegar reduced the accumulation of both hepatic cholesterol and hepatic triglyceride, and it also reduced hepatic 4-hydroxynonenal (4-HNE) lipid peroxidation. In the aortic tissues, coconut vinegar increased nitric oxide bioavailability and reduced aortic 4-HNE lipid peroxidation.

**Conclusion:**

Novel coconut vinegar is the source of antioxidants, and daily supplementation of coconut vinegar was found to attenuate dyslipidemia-induced hepatic and vascular oxidative stress by protective against cellular lipid peroxidation.

## Introduction

Hypercholesterolemia is an independent modifiable risk factor that accelerates the development of both non-alcoholic fatty liver and atherosclerosis (1). The liver and vasculature are common sites of end-organ damage during the course of dyslipidemia (2).

The rapid increase in non-alcoholic fatty liver disease (NAFLD) over the past decade, which now affects more patients than diabetes mellitus or obesity, has alarmed public health organizations worldwide (1). NAFLD has been attributed to excess consumption of dietary cholesterol (3). Having NAFLD puts the patient in danger of extrahepatic complications especially cardiovascular diseases (CVDs), which has been the leading cause of death in patients with NAFLD (2, 4). Endothelial dysfunction, an early step toward atherosclerosis, is present in NAFLD (5). NAFLD is now considered a systemic threat (6) and recognized as one of the crucial forces that drive CVDs, independent of obesity and metabolic syndrome (5, 7–9). Recent findings indicated that atherosclerotic vascular inflammation is associated with the progression of liver steatosis (10). Therefore, chronic systemic effects of dyslipidemia and interorgan communication can involve and share the course of NAFLD and atherosclerotic developments (10).

Hypercholesterolemia is a systemic disease caused by abnormal lipid metabolism (11).

In response to abnormal cholesterol load, hypercholesterolemia potentially generates a prooxidative internal milieu and subsequent onset of a chronic low-grade systemic inflammatory phenotype that linked to NAFLD and atherosclerotic pathogenesis (3, 12, 13). Hepatocellular lipotoxicity usually begins with an alteration of its endogenous lipid metabolism that drives an excess of triglycerides and various lipid species accumulations in hepatocytes (13–17). In vasculature, proatherogenic mediators such as modified low-density lipoproteins (LDLs), reactive oxygen and nitrogen species (RONS), and lipid peroxidation products directly attack membrane lipoproteins being part of their pathogenesis of atherogenic dyslipidemia (12, 18, 19). A lot of lipid peroxidative compounds, particularly aldehydes including 4-hydroxynonenal (4HNE), are involved in endothelial dysfunction by decreasing NO bioavailability (20) and arterial wall injury (21). Therefore, systemic oxidative stress is an early event of dyslipidemia that should be a target in treating or possibly preventing many dyslipidemia-associated diseases (12). Since there is no approved specific therapeutic drug to treat NAFLD or atherogenic dyslipidemia (1, 17), the only current strategy for delaying NAFLD and atherogenic dyslipidemia progression is to lower circulating lipids (1, 17). Statins are the first-line drugs for cholesterol reduction under the current guidelines for dyslipidemia (22); however they are underprescribed in NAFLD patients due to the fear of hepatotoxicity (23) and cannot cure NAFLD or atherogenic dyslipidemia (1, 17, 22). Therefore, the development of effective interventions based on the underlying pathogenesis would be desirable. To that end, one promising possibility in the effort to fight the course of NAFLD and atherogenic dyslipidemia is to look for natural products that contain relevant biological active compounds with lipid-lowering and antioxidant properties (22, 24–26).

In recent years, fruit vinegars such as apple cider vinegar have been consumed in increasing amounts for their promising therapeutic properties, which could be related to a variety of active compounds that they contain (24, 27, 28). Coconut vinegar is rich in antioxidants and has been linked to antiobesity and cholesterol reduction (26, 29, 30). Developing innovative methods and promoting applications of medicine and food homology products have become an inevitable trend (11). In the coconut milk industry, mature coconut water (MCW) is usually discarded due to its bland flavor without sweetness (31). MCW has been demonstrated to be protective against dyslipidemia and its lipid-lowering effects, which is higher than that caused by tender coconut water (TCW) (32), which makes it appealing for producing vinegar. However, the effect of coconut vinegar on end-organ damage of hepatic and vascular oxidative stress has not yet been fully explored. The purpose of the current study is to evaluate the effect of daily supplementation of coconut vinegar on serum metabolic profiles, fatty liver, and hepatic and aortic oxidative stress in high-cholesterol diet (HCD) fed rats.

## Materials and Methods

### Coconut Vinegar Preparation

Coconut vinegar was prepared by mixing coconut water from fresh, mature coconuts (*Cocos nucifera* Linn.), Prachuap Khiri Khan, Thailand with coconut sap sugar at 22° Brix. *Saccharomyces cerevisiae* TISTR 5019 from Thailand Institute of Scientific and Technological Research, Pathumthani, Thailand was used in the alcoholic fermentation and incubated at 37°C for 7 days. Mature coconut wine (15% alcohol concentration) was mixed with mature coconut water. Then, the blended coconut wine solution was fermented by *Acetobacter aceti* TISTR 354 from Thailand Institute of Scientific and Technological Research, Pathumthani, Thailand at 37°C for 4 weeks during the stage of acetic acid production. The maturing coconut vinegar was deactivated for 30 min at 55°C and filtered on Whatman No. 4 filter paper. The final products were stored at 4°C until required for further study.

### Bioactive Compounds of Coconut Vinegar

The total polyphenol content of the coconut vinegar was determined using the Folin–Ciocalteu colorimetric method with gallic acid as the standard (33). The polyphenolic compounds were determined using in-house method based on Bolivian Journal of chemistry (34) by Central Laboratory (Thailand) Co. Ltd. Organic acid by-products were analyzed using high-performance liquid chromatography with diode-array detection (HPLC/DAD) according to the standard protocol at Central Laboratory, Chiangmai, Thailand.

### Determination of Antioxidant Capacity

The 1,1-diphenyl-2-picrylhydrazyl (DPPH) radical scavenging activity assay was previously described (35). The functional properties of the coconut vinegar were analyzed to evaluate its antioxidant capacity. Briefly, 50 μL of coconut vinegar was added to 1 mL of working DPPH solution and then incubated for 30 min in the dark at room temperature. Here, 200 μM/mL of Trolox solution was used as a positive control. The absorbance of the mixture was measured at a wavelength of 517 nm. The percentage of DPPH radical scavenging activity was calculated as per the formula previously described (35).

The 2,2′-azinobis-(3-ethylbenzothiazoline-6-sulfonate) (ABTS) radical scavenging activity was determined according to the method that was previously reported (35). A total of 30 μL of the coconut vinegar was mixed with 1.5 mL of working ABTS solution, then incubated for 6 min in the dark at room temperature, and its absorbance was measured at 732 nm. The percentage of ABTS scavenging activity was determined by using 200 μM/mL of Trolox as a positive control. The ABTS scavenging activity (35) and the trolox equivalent antioxidant capacity (TEAC) were calculated based on a previous study (36).

### Animal Models

The animal protocols used in this study were approved by the Animal Ethics Committee of Naresuan University, Thailand (approval number: NU-AE620718) in accordance with the regulations of the institutional guidelines for the care and use of laboratory animals.

**Figure 1:**
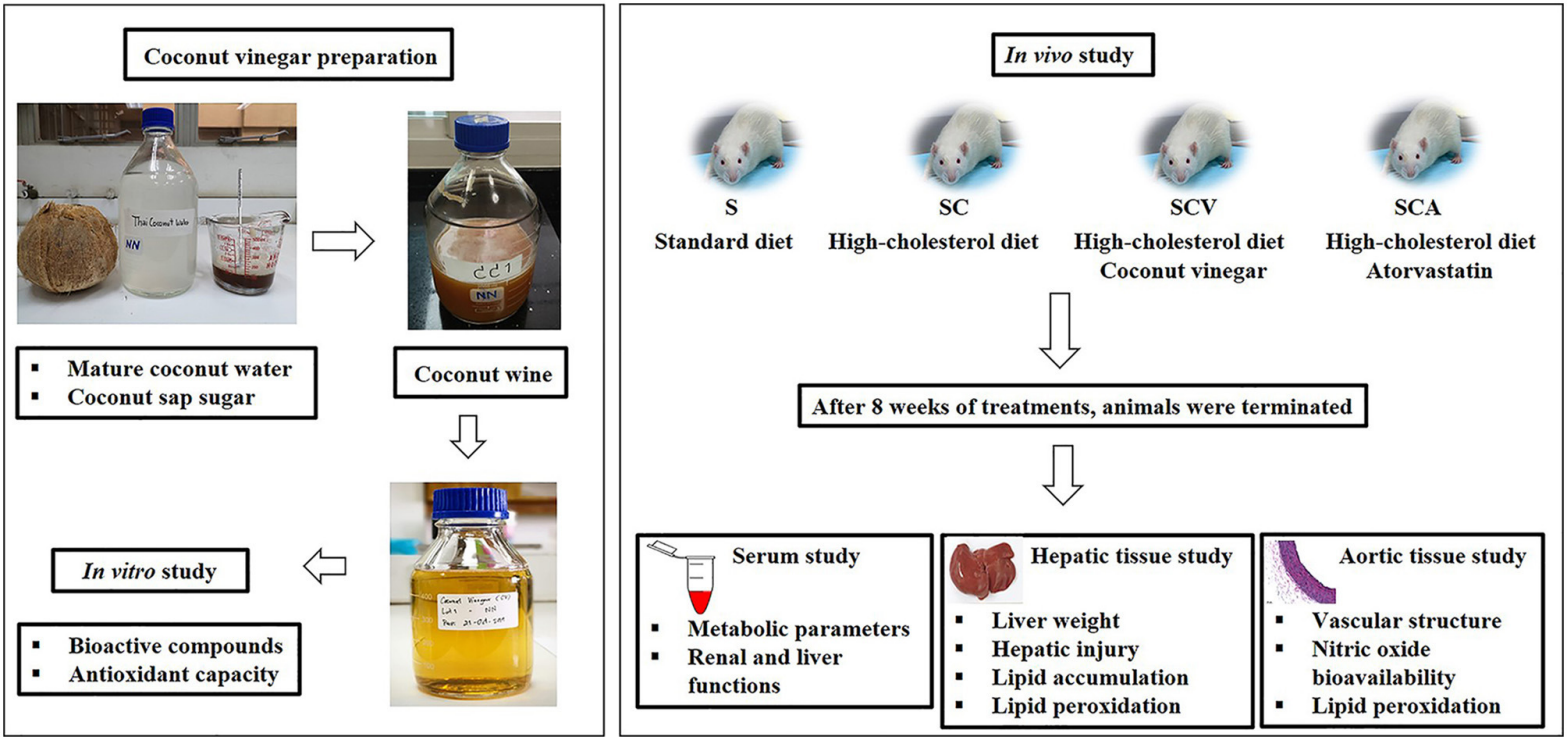
Study protocol.

Four-week-old male Sprague-Dawley rats weighing 150–200 g were obtained from Nomura Siam International Co., Ltd. Bangkok, Thailand, and housed at the Center for Animal Research, Naresuan University, Phitsanulok, Thailand, under a controlled environment (a 12 h light-dark cycle, a temperature at 25 ± 2°C. and humidity at 45–60%). They were fed with a standard diet and filtered water *ad libitum* for 1 week. The acclimatized rats were then randomly divided into 4 groups (*n* = 6) and treated for 8 weeks (Figure 1). The first group was fed a standard diet (Smart heart No. 8HT41, Complete and balance formulation; crude protein more than 24%, crude fat more than 4.5%, crude fiber less than 5%, moisture less than 10% and ash less than 10%; S) as a control. The second group was fed HCD (standard diet + 1% (w/w) of cholesterol, SC). The third group received HCD supplemented with coconut vinegar at a dose of 1 mL per kg of body weight per day (SCV). The fourth group received HCD with atorvastatin at a dose of 30 mg per kg of body weight per day, as a positive control (SCA). The rat's body weights were determined weekly, while their total food consumptions were examined daily. The coconut vinegar and the atorvastatin were administrated by oral gavage. After sacrifice, aortic tissues, perirenal fat pads, and the liver were dissected and weighed.

### Determination of Blood Biochemistry

Fasting blood sugar (FBS), total cholesterol (TC), high-density lipoprotein cholesterol (HDL-C), low-density lipoprotein cholesterol (LDL-C), triglyceride (TG), aspartate amino-transferase (AST) activity, alanine amino-transferase (ALT) activity, albumin, blood urea nitrogen (BUN), as well as creatinine (Cr) were analyzed using an automate biochemistry analyzer (Cobas c 111 analyzer, Roche Diagnostics, Basel, Switzerland).

### Evaluation of Livers and Aortae Morphology

After the tissues were fixed with 10% (v/v) formalin for 24 h, they were then embedded in paraffin and dissected into sections of 5 μm thickness. The tissue sections were rehydrated using a series of ethanol concentrations. The hematoxylin and eosin (H&E) were stained to observe the histological alterations.

For the determination of hepatic fat accumulation, frozen liver tissues were embedded in optimum cutting temperature compound (CAT.05-9801, Bio-optical, Milano, Italy), and then 8 μm sections of the frozen livers were stained with oil red O working solution (CAT. O0625, Sigma-Aldrich, USA) for 15 min at room temperature and counterstained with hematoxylin for 10 s. All images of the stained sections were visualized under a light microscope, and the alterations of the histological features were determined by using the Image-J version 1.50e.

### Analysis of Hepatic Triglycerides and Cholesterol Contents

Isolated liver samples (0.5 g) were subjected to chloroform/methanol (2:1, v/v) solution and dried under N_2_ for extracting the hepatic lipids as described by Folch et al. (37). The dried samples were dissolved, and the hepatic triglycerides and cholesterol contents were measured by using a colorimetric assay kit (Merck Millipore, Darmstadt, Germany) according to the manufacturer's protocols.

### Hepatic and Aortic Homogenizations and Protein Extractions

The frozen tissues were homogenized in ice-cold lysis buffer (phosphate buffer saline; PBS containing a protease inhibitor cocktail (CAT. 539131, Merck Millipore, Darmstadt, Germany; 1:100) by using a pestle and mortar. The tissue homogenates were centrifuged at 14,000 × g at 4°C for 10 min, which was followed by the supernatants being collected. The protein concentration was determined by using the bicinchoninic acid (BCA) protein assay kit (CAT.71285, Merck Millipore, Darmstadt, Germany) according to the manufacturer's protocols.

### Western Blot Analysis

The extracted proteins were separated using 12% of SDS-PAGE and then transferred to a polyvinylidene difluoride (PVDF) membrane, which was then blocked for 1 h in blocking solution [5% of non-fat milk in Tris-buffered saline (pH 7.4)] and incubated with a specific primary antibody at 4°C overnight [anti-4-HNE (CAT. ab46544, Abcam, 1:1,000, Cambridge, UK), anti HO-1 (CAT. ab13243, Abcam, 1:1,000, Cambridge, UK), anti-tubulin (CAT. MA5-16308, Thermo Fisher Scientific, 1:2,500, Rockford, IL, USA), and anti-GAPDH (CAT. ABS16, Merck Millipore, 1:2,500, Darmstadt, Germany)]. Subsequently, they were washed and incubated in horseradish peroxidase (HRP)-conjugated secondary antibody (CAT. AP307P, Merck Millipore, 1:5,000, Darmstadt, Germany) for 2 h at room temperature, and then the antibody–antigen complexes were visualized by HRP detection reagent (CAT.WBLUF0100, Merck Millipore, Darmstadt, Germany) and detected using the Gel Doc XR+ system (Bio-Rad Laboratories, Inc., Hercules, CA, USA). Band densities were quantified using Image Lab software version 5.2.1 (Bio-Rad Laboratories, Inc.) and normalized with housekeeping protein expression.

### Determination of Aortic Nitrate/Nitrite Contents

The total nitrate/nitrite contents were determined as an index of NO generation in aortic homogenates by using a nitrate/nitrite colorimetric assay kit (Cayman Chemical Company, Ann Arbor, MI, USA), according to the manufacturer's instructions. The absorbance was measured using a microplate reader at a wavelength of 540 nm (Synergy™ HT, Winooski, USA). The amount of total nitrate/nitrite was normalized to the protein contents of their respective aortae.

### Statistical Analysis

All the data were presented as mean ± SEM. One-way analysis of variance (ANOVA) followed by Turkey's *post-hoc* analysis being performed using GraphPad Prism 5.0. Values of *p* < 0.05 were considered statistically significant.

## Results

### Bioactive Compounds and Antioxidant Capacity

A variety of bioactive compounds were found in the coconut vinegar product. It contained the total polyphenol content at 0.16 mg gallic acid equivalent (GAE)/mL. Gallic acid, isoquercetin, and rutin, the main polyphenolic compounds in coconut vinegar, were detected at 36.64, 10.23, and 5.45 mg/kg, respectively. The metabolites by-product of organic acids during fermentation were citric acid, malic acid, tartaric acid, acetic acid, and lactic acid at the concentrations of 101.62, 72.48, 6.37 mg/kg, 2.72 g/100 g, and 332.62 mg/kg, respectively. However, undetected polyphenolic compounds and organic acids had low levels in mature coconut water. DPPH radical scavenging activity of coconut vinegar 50 μL was 43.49 ± 4.35% and ABTS^+^ radical scavenging activity of coconut vinegar 30 μl was 26.42 ± 0.62%. The antioxidant capacity of 30 μL of coconut vinegar was 181.55 ± 8.15 μM TEAC, as determined by ABTS assay as shown in Table 1.

**Table 1:** Bioactive compounds and antioxidant capacity.

**Analysis**	**Mature coconut water**	**Coconut vinegar**	**Methods**
Total polyphenol (mg GAE/mL)	0.04	0.16	The Folin–Ciocalteu colorimetric method
Polyphenolic compounds			In-house method based
Gallic acid (mg/kg)	Not detected	36.64	on Bolivian Journal of
Isoquercetin (mg/kg)	Not detected	10.23	Chemistry
Rutin (mg/kg)	Not detected	5.45	
Organic acids			HPLC/DAD
Citric acid (mg/kg)	62.27	101.62	
Malic acid (mg/kg)	Not detected	72.48	
Tartaric acid (mg/kg)	Not detected	6.37	
Acetic acid (g/100g)	202.67 (mg/kg)	2.72	
Lactic acid (mg/kg)	64.13	332.62	
Antioxidant capacity			
DPPH radical scavenging activity (%)	ND	43.49 ± 4.35	DDPH assay
ABTS+ radical scavenging activity (%)	ND	26.42 ± 0.62	ABTS assay
Trolox equivalent antioxidant capacity (TEAC)	ND	181.55 ± 8.15	ABTS assay

*ND, no data*.

### Effect of Coconut Vinegar Supplementation on Metabolic Parameters Did Not Alter Renal and Hepatic Physiological Functions

**Figure 2:**
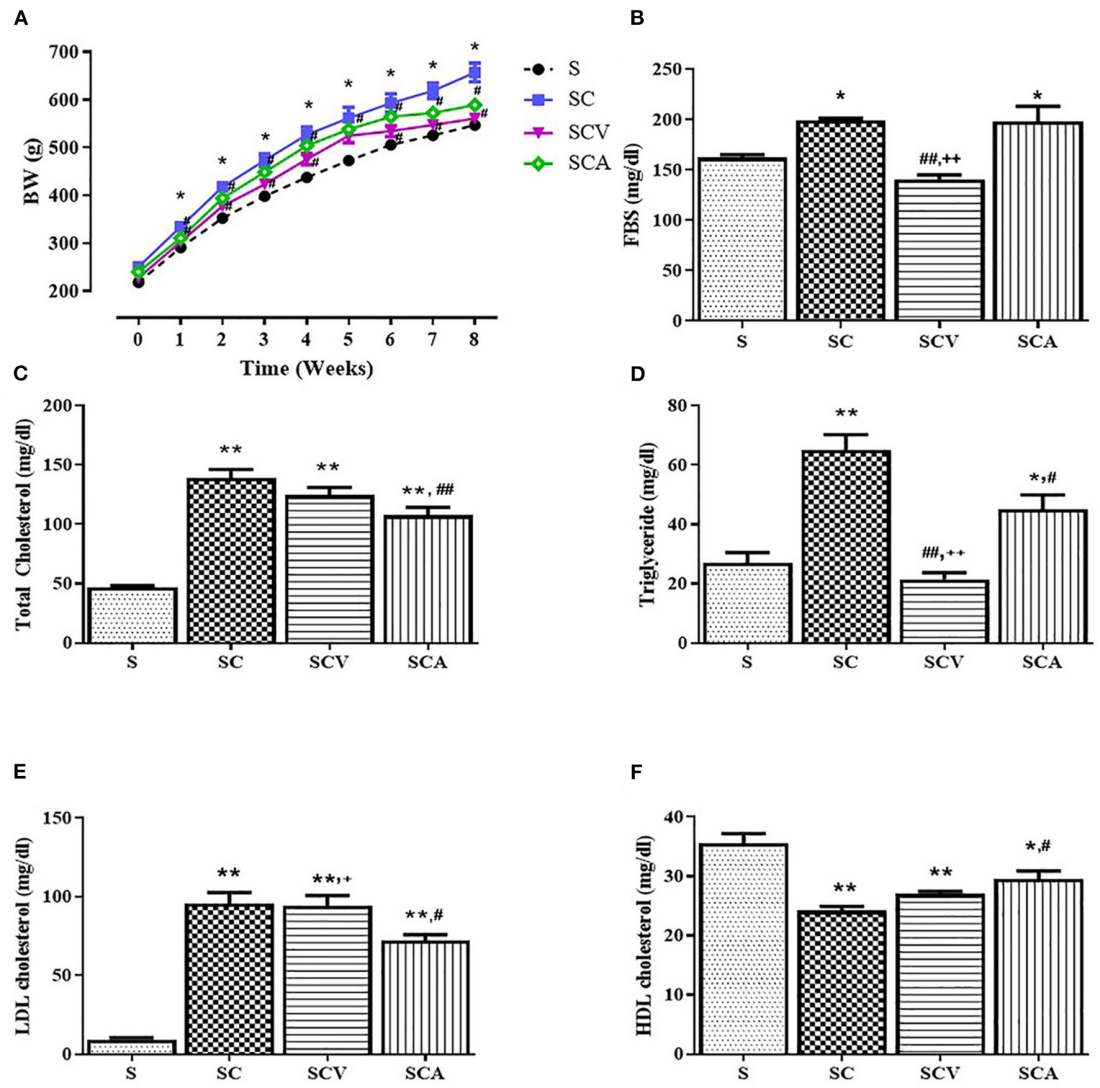
Effect of coconut vinegar supplementation on metabolic parameters. **(A)** Body weight (BW); **(B)** Fasting blood sugar (FBS); **(C)** Total cholesterol; **(D)** Triglyceride; **(E)** LDL-cholesterol; **(F)** HDL-cholesterol. Each line or bar presented in mean ± SEM. *p < 0.05; **p < 0.01 vs. the control group; ^#^*p* < 0.05; ^##^*p* < 0.05 vs. the SC group; ^+^*p* < 0.05; ^++^*p* < 0.01 vs. the SCA group (*n* = 6 per group).

The effect of coconut vinegar on the body weight of the HCD-fed rats is represented in Figure 2A. There were no significant differences in initial body weights and total food consumption (data not shown) among the groups; however, the weight of all the rats increased during the experimental period. The body weights of the rats in the HC group were significantly higher than the control group from week 2 to 8. The HCD-fed rats treated with atorvastatin displayed a lower body weight than the SC group from week 2 to 8, while the coconut vinegar-treated group showed a lower body weight than the SC groups from week 2 to 8, except for week 5. At the end of the experimental period, the HCD-fed rats treated with coconut vinegar or atorvastatin had their body weight reduced by 14.7% and 10.4%, respectively, compared to SC group, while no significant difference in body weight was observed between the SCV and SCA group.

**Figure 3:**
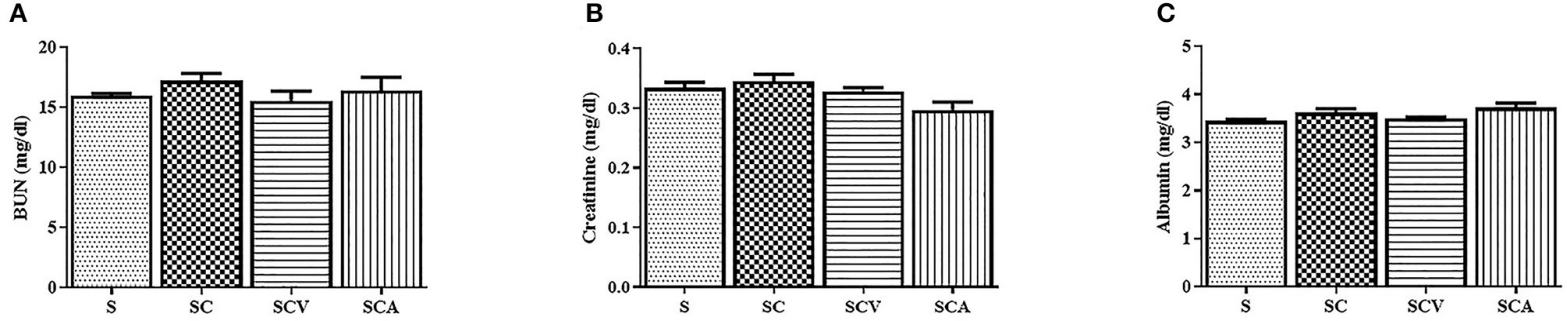
Coconut vinegar supplementation did not alter renal and hepatic physiological functions. **(A)** Blood urea nitrogen (BUN); **(B)** Creatinine; **(C)** Albumin. Each bar presented in mean ± SEM (*n* = 6 per group).

The SC group significantly increased the plasma concentrations of TC, TG, and LDL-c, while at the same time significantly decreasing the HDL levels compared to the control group (Figures 2C–F). There was a significance reduction of fasting blood sugar in the SCV group when compared with the SC group (140 ± 5 mg/dL vs. 199 ± 2 mg/dL, *p* < 0.01) (Figure 2B). The hypercholesterolemic rats treated with coconut vinegar or atorvastatin had significantly reduced plasma levels of TG when compared with HCD-fed rats (22 ± 2 mg/dL, 45 ± 5 mg/dL vs. 65 ± 5 mg/dL, *p* < 0.01 and *p* < 0.05, respectively) (Figure 2D). The decrease of the TG plasma level was more significant in the coconut vinegar-treated group than the atorvastatin-treated one. Compared to the untreated SC rats, the atorvastatin-treated ones had significantly reduced plasma LDL-C levels and increased plasma HDL-C levels (all group *p* < 0.05). However, there were no significant differences observed in LDL-c and HDL-c levels in the SCV group compared to SC group. Plasma levels of BUN, creatinine, and albumin were measured to investigate the effect of coconut vinegar on renal and hepatic physiological functions. As shown in Figures 3A–C, no significant changes were detected in the plasma levels of BUN, creatinine, and albumin among the control, SC, and the experimental groups.

### Coconut Vinegar Supplementation Reduced High Cholesterol Induced-Hepatic Injury and Perirenal Fat Pad Weight

**Figure 4:**
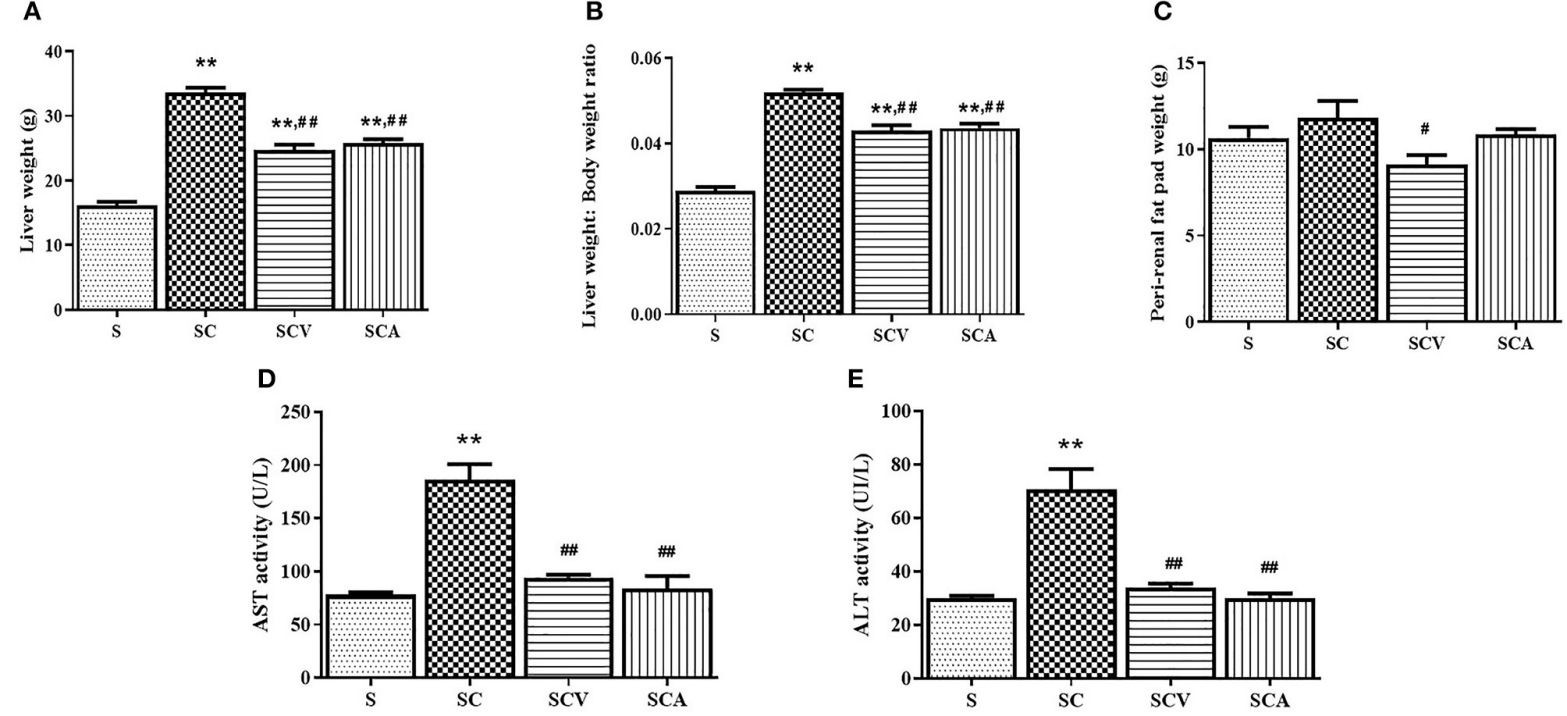
Coconut vinegar supplementation reduced high cholesterol induced-hepatic injury and perirenal fat pad weight. **(A)** Liver weight; **(B)** Liver weight to body weight ratio; **(C)** Perirenal fat pad weight; **(D)** Aspartate amino-transferase (AST) activity; **(E)** Alanine aminotransferase (ALT) activity. Each bar presented in mean ± SEM. **p < 0.01 vs. the control group; ^#^*p* < 0.05; ^*##*^*p* < 0.01 vs. the SC group (*n* = 6 per group).

The liver weight and the ratio of liver to body weight showed a significant increase in the SC group compared to the control group. However, they were reduced of both the SCV and SCA groups when compared to the SC group (24.8 ± 0.8 g, 25.8 ± 0.6 g vs. 33.6 ± 0.8 g, *p* < 0.01 and *p* < 0.01 for the liver weight; 0.04 ± 0.001, 0.04 ± 0.001 vs. 0.05 ± 0.001, all *p* < 0.01 for the ratio of liver to body weight, respectively) (Figures 4A,B). In addition, the HCD-fed rats showed a slightly higher perirenal fat pad weight than the control groups, although such elevation was not significant (*p* > 0.05) (Figure 4C). However, the perirenal fat pad weight showed a significant decrease of 22.8% in the SCV group when compared to the SC group, whereas the fat pad was unaffected by the atorvastatin treatment. The SC group had a significant elevation of both AST and ALT activities when compared with the control group (Figures 4D,E), but the levels in both the SCV and SCA groups decreased significantly when compared to the SC group, and was close to the levels observed in the control group.

### Coconut Vinegar Supplementation Reduced Hepatic Lipid Accumulation

**Figure 5:**
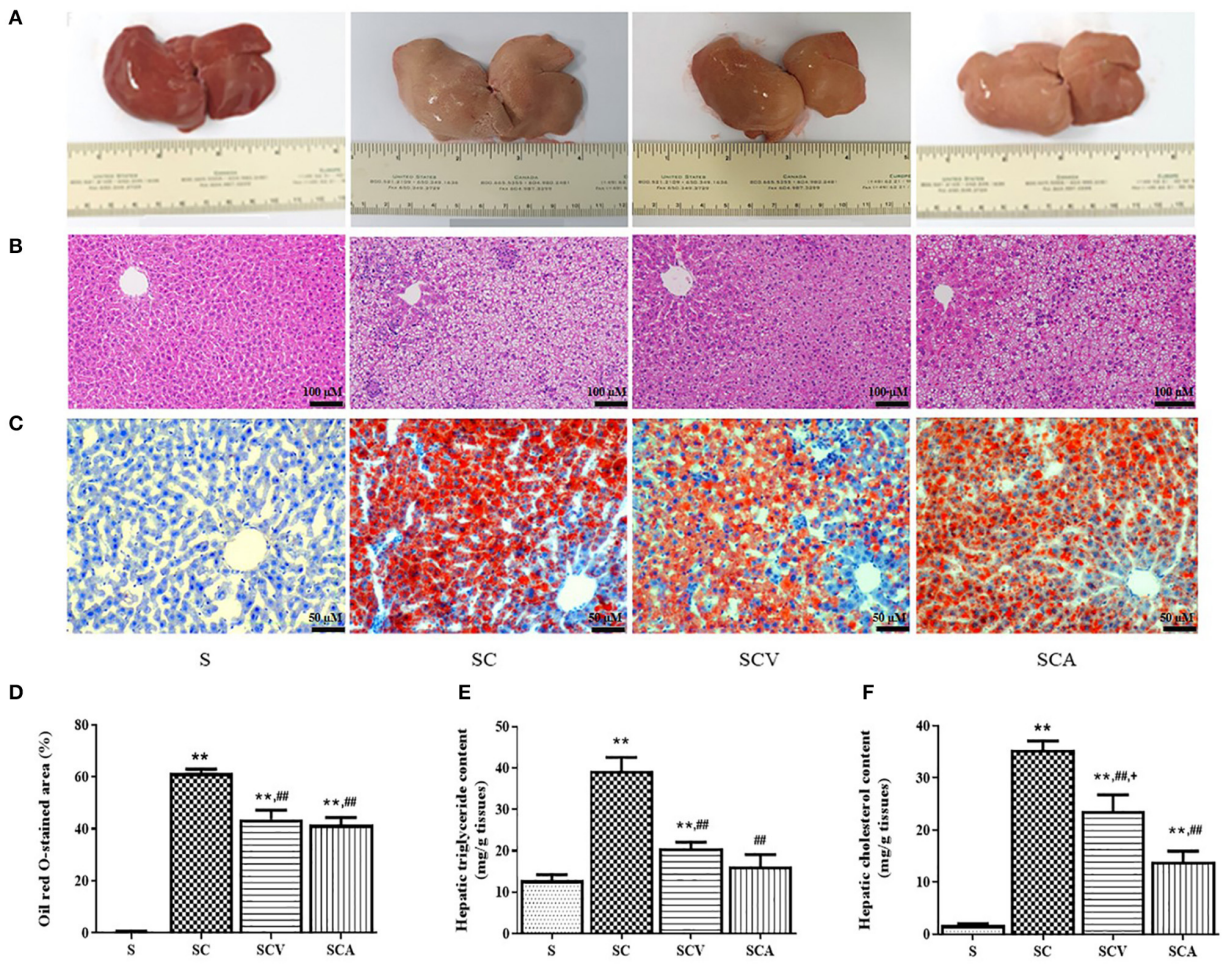
Coconut vinegar supplementation reduced hepatic lipid accumulation. **(A)** Liver morphology; **(B)** Hepatic H&E staining; **(C)** Hepatic Oil red O staining; **(D)** Oil red O stained-area; **(E)** Hepatic triglyceride content; **(F)** Hepatic cholesterol content. Each bar presented in mean ± SEM. **p < 0.01 vs. the control group; ^*##*^*p* < 0.01 vs. the SC group; ^+^*p* < 0.05 vs. the SCA group (*n* = 6 per group).

As shown in Figure 5A, the appearance of the external livers of the control rats were dark red, soft, sharp-edged, and smooth surfaces, while the ones from the rat fed HCD were yellow, larger, and greasy. The color and texture of the livers in the coconut vinegar- and atorvastatin-treated groups were improved when compared to SC group. The H&E staining (Figure 5B) of the liver sections from the control group revealed the normal histological structure, without any obvious steatosis and inflammation, but the rats fed the HCD exhibited severe steatosis with cytoplasmic lipid vacuoles and infiltration of inflammatory cells in the liver tissues. Oil Red O staining (Figure 5C) also exhibited an increased amount of Oil Red O-stained lipids in the liver sections of the SC group compared to the control group (Figure 5D). However, coconut vinegar and atorvastatin treatments significantly decreased the accumulation of intracellular lipid droplets and infiltration of inflammatory cells as well as the percentage of the Oil Red O-stained area in the HCD-fed rats. Hepatic TG and TC contents were increased in the HC group compared with the control group (39.3 ± 3.3 mg/g tissue vs. 12.8 ± 1.4 mg/g tissue, *p* < 0.01 for TG; 35.4 ± 2.0 mg/g tissue vs. 1.7 ± 0.3 mg/g tissue, *p* < 0.01 for TC, respectively). In the SCV and SCA, these levels were decreased compared to the SC group (20.6 ± 1.4 mg/g tissue, 16.2 ± 2.8 mg/g tissue vs. 39.3 ± 3.3 mg/g tissue, all *p* < 0.01 for TG; 23.7 ± 3.0 mg/g tissue, 13.9 ± 1.9 mg/g tissue vs. 35.4 ± 2.0 mg/g tissue, all *p* < 0.01 for TC, respectively) (Figures 5E,F). These effects of decreased hepatic contents of TG and TC were more significant in the atorvastatin-treated group than coconut vinegar-treated group.

### Coconut Vinegar Supplementation Reduced Hepatic Lipid Peroxidation

**Figure 6:**
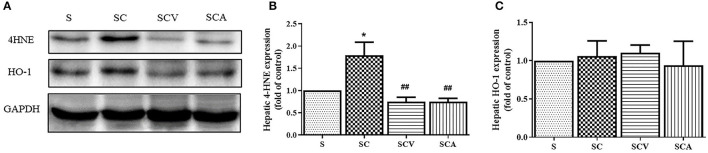
Coconut vinegar supplementation reduced hepatic lipid peroxidation. **(A)** The band of glyceraldehyde 3-phosphate dehydrogenase (GAPDH), 4-hydroxynonenal (4-HNE), and Heme oxygenase-1 (HO-1) expressions of hepatic tissues, the proteins expression was determined by western blot analysis in five experiments with independent preparations; **(B)** Quantified levels of hepatic 4-HNE expression; **(C)** Quantified levels of hepatic HO-1 expression. Each bar presented in mean ± SEM. *p < 0.05 vs. the control group; ^*##*^*p* < 0.01 vs. the SC group (*n* = 5 per group).

As shown in Figure 6, the protein expression of 4-HNE, a lipid peroxidation marker, was significantly increased in the liver of the SC group when compared with the control group (Figures 6A,B). Additionally, coconut vinegar or atorvastatin administration decreased the 4-HNE level in the liver tissues of the HCD-fed rats, compared with the SC group (all *p* < 0.05; Figure 6B), whereas the hepatic expressions of HO-1 showed that no differences existed among the four groups (all *p* > 0.05; Figure 6C).

### Effect of Coconut Vinegar Supplementation on Aortic Histopathological Changes

**Figure 7:**
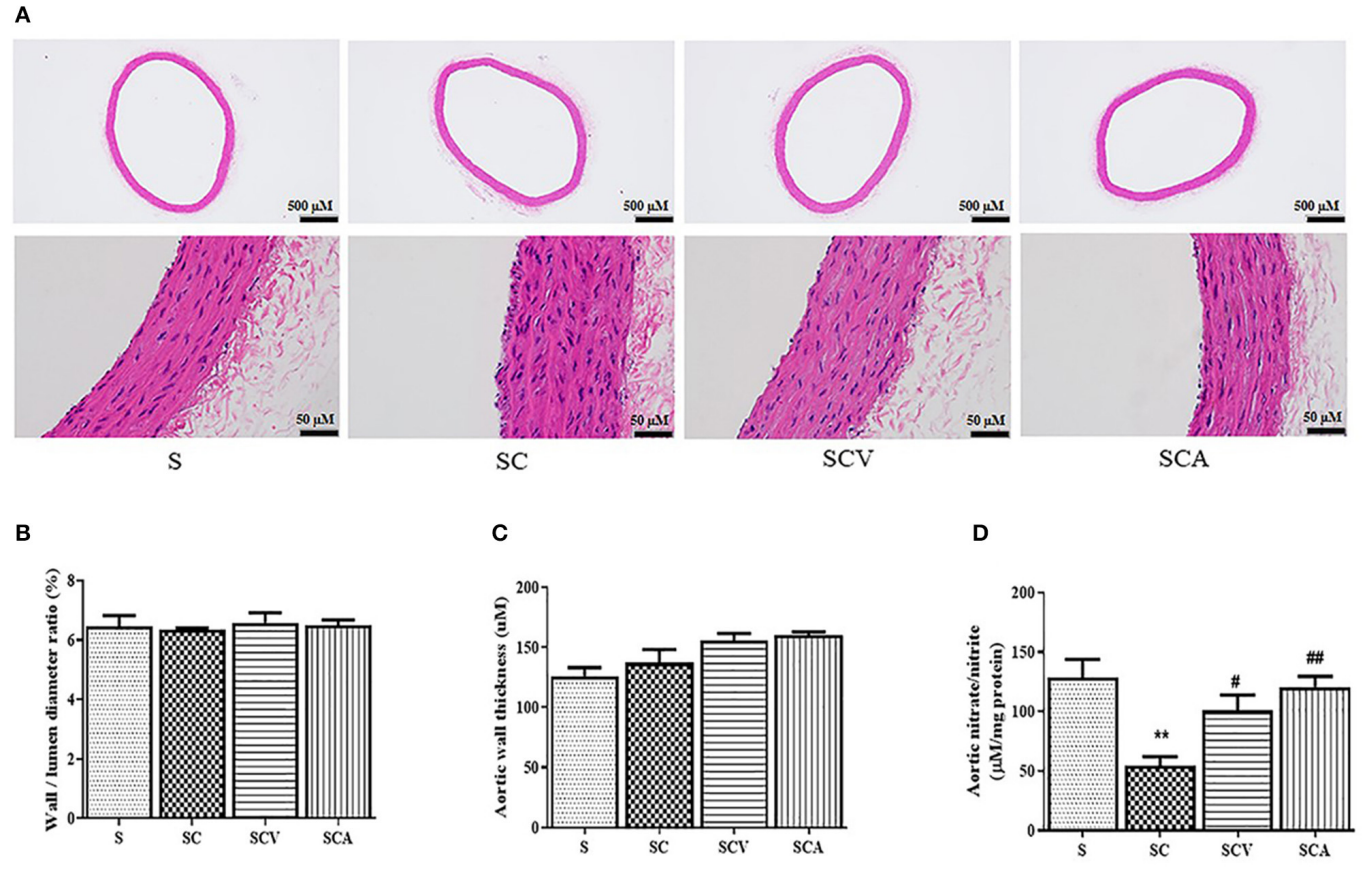
Effect of coconut vinegar supplementation on aortic histopathological changes and nitric oxide bioavailability. **(A)** Aortic H&E staining; **(B)** Aortic wall to lumen diameter ratio; **(C)** Aortic wall thickness; **(D)** Aortic nitrate/nitrite. Each bar presented in mean ± SEM. **p < 0.01 vs. the control group; ^#^*p* < 0.05; ^*##*^*p* < 0.01 vs. the SC group (*n* = 6 per group).

The histological changes of the aorta tissues were determined among the experimental groups. Representative H&E staining images of each group are shown in Figure 7A. H&E staining of the aortic sections of the control rats showed normal structure of the endothelium and smooth muscle cells, including regularly shaped and arranged endothelial cells. However, the aortic tissues exhibited remarkable histopathological changes in the SC group, showing a partial exfoliation of endothelial cells, an apparent increase in the thickness of the subendothelial layers, and a disorientation of the smooth muscle cells. However, these histopathological changes of the aortic tissues were slightly improved in the coconut vinegar- or atorvastatin-treated group. The aortic wall to the lumen diameter ratio and the aortic wall thickness in all the experimental groups showed no significant differences (Figures 7B,C).

### Effect of Coconut Vinegar Supplementation on Aortic Nitric Oxide Bioavailability

Since nitrate and nitrite contents might be considered as inert end products from nitric oxide generation and they reflected its activity; therefore, the aortic total nitrate and nitrite contents were measured to estimate the nitric oxide bioactivity. After 8 weeks, the HCD-fed rats showed both reduced aortic total nitrate and nitrite levels compared to the control group (54.6 ± 7.4 μM/mg protein vs. 128.7 ± 15.2 μM/mg protein, *p* < 0.01, respectively) (Figure 7D). A daily supplementation of coconut vinegar or atorvastatin to the HCD-fed rats increased total nitrate and nitrite levels compared to the SC group (100.7 ± 13.2 μM/mg protein, 120.6 ± 8.9 μM/mg protein vs. 54.6 ± 7.4 μM/mg protein, *p* < 0.05 and *p* < 0.01, respectively) (Figure 7D).

### Coconut Vinegar Supplementation Reduced Vascular Lipid Peroxidation

**Figure 8:**
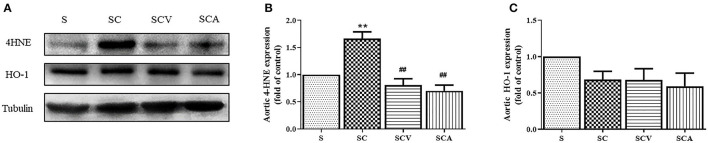
Coconut vinegar supplementation reduced vascular lipid peroxidation. **(A)** The band of glyceraldehyde 3-phosphate dehydrogenase (GAPDH), 4-hydroxynonenal (4-HNE), and Heme oxygenase-1 (HO-1) expressions of aortic tissues, the proteins expression was determined by western blot analysis in five experiments with independent preparations; **(B)** Quantified levels of aortic 4-HNE expression; **(C)** Quantified levels of aortic HO-1 expression. Each bar presented in mean ± SEM. **p < 0.01 vs. the control group; ^*##*^*p* < 0.01 vs. the SC group (*n* = 5 per group).

As shown in Figure 8, the protein expression of 4-HNE, a lipid peroxidation marker, was significantly upregulated in the aortae of the SC group when compared with the control group (Figures 8A,B). Additionally, coconut vinegar or atorvastatin administration decreased the 4-HNE expression in the aortic tissues of the HCD-fed rats compared with the SC group (all *p* < 0.05; Figures 8A,B), whereas the aortic HO-1 levels showed that no differences existed among the four groups (all *p* > 0.05; Figures 8A,C).

## Discussion

The novel coconut vinegar in this study contained a source of polyphenols and organic acids, with its dominant biological functions being relevant to its lipid-lowering effect and antioxidative stress. Daily supplementation of coconut vinegar reduced weight gain and serum triglycerides, fasting blood sugar, and fatty liver in the HCD fed rats. The key cellular mechanism of the vinegar is to protect against dyslipidaemia-induced hepatic and vascular oxidative stress by attenuating cellular lipid peroxidation.

Coconut sap sugar was used for the first time as a raw material sweetener for making coconut vinegar. Both the mature coconut water and the coconut sap sugar are derived from a variety of bioactive metabolites during fermentation (25, 26). In an industrial vinegar processing, the transition from wine to vinegar is caused by a major loss of antioxidant phenolic compounds (38). By the innovative ideas for making our coconut vinegar, a short period of microbe enzymatically fermentation with a light alcohol content of wine was to ensure that macromolecules and microbial metabolic processes are sufficient to protect active microbial population constant over the period of fermentation (39) and to preserve the metabolite by-products (31). The concentration of acetic acid is 2.72 g/100g in which actual category is fruit vinegar (40). The major concern of acidity in traditional vinegar consumption caused irritation bowel syndrome (41) and dental erosion (42). Lowering acetic acid level in vinegar could possibly be beneficial and cause fewer health concerns. Not only does acetic acid act as the main bioactive compound, but also gallic acid, isoquercetin, rutin, malic acid, and tartaric acid contained in coconut vinegar are reported to improve blood lipids and glycemic tolerance (24, 43–49). While its antioxidant capacity was at a high level compared to other fruit vinegars (24), this has heightened the active compounds and biological functions in the current vinegar product. Accordingly, raw materials and vinegar processing should be carefully considered to strengthen its biological properties.

In our finding, the HCD-fed rat showed a common feature of dyslipidemia. Daily coconut vinegar supplement in the HCD-fed rats did aggressively reduce the serum level of triglycerides, which produced the outcome of being close to normal control. The major influence of our coconut vinegar on the triglyceride serum was similar to the one fed to obese mice (26). This indicates that coconut vinegar has an anti-hypertriglyceridemia dominant, while superior benefits of serum TC, LDL-C, and HDL-C reduction is due to atorvastatin in the HCD model. By affinal drug and diet, coconut vinegar is used rationally as a medicine and food homology product (50). Recent studies of HCD-fed rats reported that mature coconut water, a main, material of our vinegar caused a reduction of hepatic lipogenic enzyme activities and increased the hepatic bile acid, fecal bile acids, and neutral sterols excretion (32, 51). Beneficial effects of mature coconut water feeding or coconut vinegar supplementation are in a similar trend to serum and tissue lipid parameter reductions (32). While the antioxidant capacity of bioactive by-products in coconut vinegar, as shown in Table 1, was superior to mature coconut water, the biological function of the coconut vinegar reduced dyslipidemia, which may related to the lipid metabolism pathways. Therefore, the bioactivity of coconut vinegar on the metabolism of lipid in the liver requires further investigation.

The HCD-fed rats demonstrated greater hepatic weight, pale-yellow color with small and large droplets of fat deposited in the hepatocytes and excess perirenal fat pads implied a severe steatosis and an increased visceral fat deposition. An elevation of intracellular fat accumulation may cause weight gain. A daily supplementation of coconut vinegar demonstrated limited weight gains as well as reduced liver weights. Its bioactivity also showed a reduction of hepatic cholesterol and hepatic triglycerides accumulation as well as a significant reduction in perirenal adipose tissues. This suggested that the biological function of coconut vinegar contributes to visceral fat distribution. The reduction of fat deposition in the extra-adipose tissues reflects a secondary impact of the coconut vinegar supplementation, which reduces obesity caused by dyslipidemia. In this study, controlling weight gain which is caused by coconut vinegar could be related to the metabolic pathways rather than the loss of appetite because their total food consumptions remain unchanged. Additionally, Yamashita et al. reported that acetic acid improved lipid metabolism in both the skeletal muscles and adipose tissues, and reduced the potential of obesity in type-2 diabetic rats (48). Gallic acid in coconut vinegar received a lot of attention regarding its potent antidiabetic properties (43, 49). It is able to preserve pancreatic tissue, promote insulin secretion, reduce gluconeogenesis, and also induce the expression of glucose transporter protein type-4 (GLUT4) in the insulin-responsive tissues (43, 49). This suggested that acetic acid and gallic acid found in coconut vinegar could be active compounds toward antiobesity and boosted glycemic control.

Altered lipid metabolism that occurs in NAFLD causes an abnormal elevation of free fatty acids, cholesterol, and triacylglycerols which accelerates the accumulation of fat (15, 17) and the overproduction of free radicals in hepatocytes (13). The metastable products of reactive species reaction such as aldehydes are intensively cytotoxic, which leads to amplified oxidative stress in hepatocytes during the progression of NAFLD (13–15). An overwhelm of highly reactive species in the liver will spill over into the circulation, which extends the oxidative stress to the systemic circulation (1). The increase in circulating oxidizing species can further modify circulating metabolites and phospholipids of the plasma membranes to generate oxidizing pathogenic molecules including ox-LDL (1, 3). These dysfunctional metabolites mainly contribute to endothelial dysfunction and low-grade systemic inflammation that are placed on more susceptible CVDs (2, 3). The 4-hydroxy 2-nonenal (4-HNE) is the most toxic and abundant metastable aldehyde product of lipid peroxidation (13). Therefore, 4-HNE can be an early indicator of cellular oxidative damage and a key active player that increases the vulnerability of atherogenic dyslipidemia in NAFLD (1, 13, 15). In HCD-fed rats, the coconut vinegar effect was able to protect against end-organ lipid peroxidation. The reduction of 4-HNE in hepatic and aortic tissues was possibly caused by its lowering circulating lipid and modulating lipotoxicity effects in these tissues. Coconut vinegar modified oxidation which was due to its biological antioxidant capacity, rather than contributing to the increase of the defensive mechanism of an endogenous antioxidant enzyme, such as the major ARE-antioxidant enzyme and heme oxygenase-1 (HO-1) (52), suggested that daily coconut vinegar supplementation exerted an antioxidative efficiency on steatosis and atherogenic dyslipidemia, which served as a potential therapeutic targeting on lipid peroxidation. However, protective effects and molecular mechanisms of coconut vinegar on prooxidant–antioxidant balance and advanced oxidation protein products in the evolution of hyperlipidemia should be further investigated.

This study suggests dietary coconut vinegar supplementation may decrease the risk of developing NAFLD and atherogenic dyslipidemia by reverse of intracellular lipid accumulation from non-adipocyte tissues and clearance of triglycerides from the circulation. However, the experiment was designed to supplement coconut vinegar or atorvastatin at the time of HCD feeding before the initiation of the disease. This histological study showed that the liver was readily damaged due to it being a sensitive end-organ, whereas the aortic tissues showed insignificant structural alterations in the HCD-fed rats. The administration of atorvastatin can limit serum lipid alterations but is unable to prevent fat accumulation and cellular lipid peroxidation in the HCD-fed rats. Our findings suggested that fatty liver and vascular oxidative stress requires early intervention and medication management after dyslipidemia has occurred, and it would be too late to prevent secondary NAFLD and CVDs adverse outcomes. It was found that individual intervention of coconut vinegar or atorvastatin was unsuccessful in completely reversing hepatic steatosis and cellular lipid peroxidation. Therefore, a dual supplementation of low-doses of atorvastatin and coconut vinegar requires further investigation in the prevention of dyslipidemia-related complications. This strategy would offer therapeutic potential for individuals who are at risk from dyslipidemia. According to the integrative actions of the bioactive compounds in coconut vinegar and its multiple health benefits without toxicity, the incorporation of it into daily meals of healthy people or home-based intervention clinical trials, would be tempting. The biological properties of coconut vinegar offer an alternative to dyslipidemia patients, especially the statin intolerance ones, but also to high-risk individuals with hepatic steatosis and cardiovascular diseases.

## Conclusion

Coconut vinegar is rich in polyphenols and bioactive organic acids. Daily coconut vinegar supplementation improved lipid profile, glycemic control, and hepatic lipid accumulation in the HCD-fed rats. Protection against cellular oxidative stress demonstrated a key biological function of coconut vinegar. Antioxidative effects of coconut vinegar in the modulation of lipid oxidation protects hepatic and vascular damage. Therefore, coconut vinegar is a potential health-related compound of natural source of antioxidants.

## Data Availability Statement

The raw data supporting the conclusions of this article will be made available by the authors, without undue reservation.

## Ethics Statement

The animal study was reviewed and approved by Animal Ethics Committee of Naresuan University, Thailand (approval number: NU-AE620718).

## Author Contributions

SA, SK, WM, and NN: conceptualization. NJ, PS, SK, WM, and NN: methodology and data curation. SK, WM, and NN: formal analysis and review and editing the manuscript. PS and WM: writing original draft. All authors contributed to the article and approved the submitted version.

## Funding

This work was supported by Biodiversity-Based Economy Development Office (Public Organization) and National Research Council of Thailand, grant number BEDO-NRCT 48/2562.

## Conflict of Interest

The authors declare that the research was conducted in the absence of any commercial or financial relationships that could be construed as a potential conflict of interest.

## Publisher's Note

All claims expressed in this article are solely those of the authors and do not necessarily represent those of their affiliated organizations, or those of the publisher, the editors and the reviewers. Any product that may be evaluated in this article, or claim that may be made by its manufacturer, is not guaranteed or endorsed by the publisher.

## Abbreviations

NAFLD: Non-alcoholic fatty liver disease
CVDs: Cardiovascular diseases
RONS: Reactive oxygen and nitrogen species
4-HNE: 4-Hydroxynonenal
SOS: Systemic oxidative stress
MCW: Mature coconut water
S: Standard diet (control)
SC: Standard diet + 1% cholesterol
SCV: Standard diet + 1% cholesterol supplemented with coconut vinegar
SCA: Standard diet + 1% cholesterol supplemented with atorvastatin
DPPH, 1: 1-diphenyl-2-picrylhydrazyl
ABTS, 2: 2′-azinobis-(3-ethylbenzothiazoline-6-sulfonate)
TEAC: Trolox equivalent antioxidant capacity
HCD: High-cholesterol diet
FBS: Fasting blood sugar
TC: Total cholesterol
HDL-C: High-density lipoprotein cholesterol
LDL-C: Low-density lipoprotein cholesterol (LDL-C)
TG: Triglyceride
AST: Aspartate amino-transferase
ALT: Alanine amino-transferase
BUN: Blood urea nitrogen (BUN)
Cr: Creatinine
HO-1: Heme Oxygenase-1.
