# Supplementary material for: Novel Coconut Vinegar Attenuates Hepatic and Vascular Oxidative Stress in Rats Fed a High-Cholesterol Diet
Source: Front Nutr. 2022 Mar 9;9:835278. doi: 10.3389/fnut.2022.835278 (PMC8959456; doi:10.3389/fnut.2022.835278)
Supplement: Supplementary file 2 [file Data_Sheet_2.PDF]

บริษัท ห้องปฏิบัติการกลาง (ประเทศไทย) จำกัด
Central Laboratory (Thailand) Co.,Ltd.
สาขาเชียงใหม่ : 164/86 หมู่ที่ 3 ต.ดอนแก้ว อ.แม่ริม จ.เชียงใหม่ 50180
Chiangmai Branch : 164/86 Moo 3 Donkaew, Maerim, Chiangmai 50180 Thailand
Tel : (66) 0 5389 6131, (66) 0 5389 6133 Fax : (66) 0 5389 6052, (66) 0 5389 6131 ต่อ 705
http://www.centrallabthai.com

Central Lab
One Stop & Fast Services

รายงานผลการทดสอบ

Mature Coconut Water

วันที่ออกรายงาน 25 มิถุนายน 2563

เลขที่รายงาน TRCM63/15996

ชื่อและที่อยู่ลูกค้า โครงการบูรณาการการใช้ประโยชน์จากความหลากหลายทางชีวภาพมะพร้าวไทยเพื่อการสร้างพันธุ์มะพร้าวมูลค่าทางเศรษฐกิจสูงและการเพิ่มมูลค่าผลิตภัณฑ์มะพร้าว (เป็นโครงการที่ 2)
ภาควิชาพืชไร่นา คณะเกษตร มหาวิทยาลัยเกษตรศาสตร์ วิทยาเขตกำแพงแสน
ต. กำแพงแสน อ. กำแพงแสน จ. นครปฐม 73140

รายละเอียดตัวอย่าง (ข้อมูลจากลูกค้า) น้ำมะพร้าวแกง

รหัสตัวอย่าง CM63/04926-002

ลักษณะและสภาพตัวอย่าง ประเภทตัวอย่าง : น้ำมะพร้าว
ภาชนะบรรจุ : หลอดพลาสติก ปิดสนิท, จำนวน : 10 หลอด, น้ำหนัก/ปริมาตร : 50 มิลลิลิตร/หลอด
อุณหภูมิขณะรับ : แช่เย็น, สภาพตัวอย่างปกติ

วันที่รับตัวอย่าง 28 พฤษภาคม 2563

วันที่ทดสอบ 01 มิถุนายน 2563 - 24 มิถุนายน 2563

ผลการทดสอบ

รายการทดสอบ	ผลการทดสอบ	หน่วย	LOD	วิธีทดสอบอ้างอิง
**Fatty acid composition**				
Butyric acid (C4:0)	Not Detected	g/100g	0.01	In house method TE-CH-208 based on AOAC (2012) 996.06
Caproic acid (C6:0)	Not Detected	g/100g	0.01	
Caprylic acid (C8:0)	0.02	g/100g	-	
Capric acid (C10:0)	0.01	g/100g	-	
Undecanoic acid (C11:0)	Not Detected	g/100g	0.01	
Lauric acid (C12:0)	0.13	g/100g	-	
Tridecanoic acid (C13:0)	Not Detected	g/100g	0.01	
Myristic acid (C14:0)	0.07	g/100g	-	
Pentadecanoic acid(C15:0)	Not Detected	g/100g	0.01	
Palmitic acid (C16:0)	0.05	g/100g	-	
Heptadecanoic acid (C17:0)	Not Detected	g/100g	0.01	
Stearic acid (C18:0)	0.02	g/100g	-	
Arachidic acid (C20:0)	Not Detected	g/100g	0.01	

รายงานฉบับนี้มีผลเฉพาะกับตัวอย่างที่นำมาทดสอบเท่านั้น
รายงานผลการทดสอบต้องไม่ถูกทำสำเนาเฉพาะเพียงบางส่วน โดยไม่ได้รับความยินยอมเป็นลายลักษณ์อักษรจากห้องปฏิบัติการ ยกเว้นทำทั้งฉบับ

บริษัท ห้องปฏิบัติการกลาง (ประเทศไทย) จำกัด
Central Laboratory (Thailand) Co.,Ltd.
สาขาเชียงใหม่ : 164/86 หมู่ที่ 3 ต.ดอนแก้ว อ.แม่ริม จ.เชียงใหม่ 50180
Chiangmai Branch : 164/86 Moo 3 Donkaew, Maerim, Chiangmai 50180 Thailand
Tel : (66) 0 5389 6131, (66) 0 5389 6133 Fax : (66) 0 5389 6052, (66) 0 5389 6131 ต่อ 705
http://www.centrallabthai.com

รายงานผลการทดสอบ

Mature Coconut Water

วันที่ออกรายงาน 25 มิถุนายน 2563
เลขที่รายงาน TRCM63/15996

ผลการทดสอบ

รายการทดสอบ	ผลการทดสอบ	หน่วย	LOD	วิธีทดสอบอ้างอิง
Heneicosanoic acid(C21:0)	Not Detected	g/100g	0.01	
Behenic acid (C22:0)	Not Detected	g/100g	0.01	
Tricosanoic acid (C23:0)	Not Detected	g/100g	0.01	
Lignoceric acid (C24:0)	Not Detected	g/100g	0.01	
Saturated fat	0.30	g/100g	-	
Myristoleic acid (C14:1)	Not Detected	g/100g	0.01	
cis-10-Pentadecenoic acid(C15:1n10)	Not Detected	g/100g	0.01	
Palmitoleic acid (C16:1n7)	Not Detected	g/100g	0.01	
cis-10-Heptadecenoic acid(C17:1n10)	Not Detected	g/100g	0.01	
Trans-9-Elaidic acid (C18:1n9t)	Not Detected	g/100g	0.01	
cis-9-Oleic acid (C18:1n9c)	0.03	g/100g	-	
cis-11-Eicosenoic acid(C20:1n11)	Not Detected	g/100g	0.01	
Erucic acid (C22:1n9)	Not Detected	g/100g	0.01	
Nervonic acid (C24:1n9)	Not Detected	g/100g	0.01	
Monounsaturated fatty acid	0.03	g/100g	-	
trans-Linolelaidic acid(C18:2n6t)	Not Detected	g/100g	0.01	
cis-9,12-Linoleic acid (C18:2n6)	Not Detected	g/100g	0.01	
gamma-Linolenic acid (C18:3n6)	Not Detected	g/100g	0.01	
alpha-Linolenic acid (C18:3n3)	Not Detected	g/100g	0.01	
cis-11,14-Eicosadienoic acid (C20:2)	Not Detected	g/100g	0.01	
cis-8,11,14-Eicosatrienoic acid (C20:3n6)	Not Detected	g/100g	0.01	
cis-11,14,17-Eicosatrienoic acid (C20:3n3)	Not Detected	g/100g	0.01	
Arachidonic acid (C20:4n6)	Not Detected	g/100g	0.01	
cis-13,16-Docosadienoic acid(C22:2)	Not Detected	g/100g	0.01	
cis-5,8,11,14,17-Eicosapentaenoic acid(C20:5n3)	Not Detected	g/100g	0.01	

รายงานฉบับนี้มีผลเฉพาะกับตัวอย่างที่นำมาทดสอบเท่านั้น
รายงานผลการทดสอบต้องไม่ถูกทำสำเนาเฉพาะเพียงบางส่วน โดยไม่ได้รับความยินยอมเป็นลายลักษณ์อักษรจากห้องปฏิบัติการ ยกเว้นทำทั้งฉบับ

Central Laboratory (Thailand) Co.,Ltd.
บริษัท ห้องปฏิบัติการกลาง (ประเทศไทย) จำกัด
Central Laboratory (Thailand) Co.,Ltd.
สาขาเชียงใหม่ : 164/86 หมู่ที่ 3 ต.ดอนแก้ว อ.แม่ริม จ.เชียงใหม่ 50180
Chiangmai Branch : 164/86 Moo 3 Donkaew, Maerim, Chiangmai 50180 Thailand
Tel : (66) 0 5389 6131, (66) 0 5389 6133 Fax : (66) 0 5389 6052, (66) 0 5389 6131 ต่อ 705
http://www.centrallabthai.com

รายงานผลการทดสอบ

Mature Coconut Water

วันที่ออกรายงาน 25 มิถุนายน 2563
เลขที่รายงาน TRCM63/15996
หน้า 03/04

ผลการทดสอบ

รายการทดสอบ	ผลการทดสอบ	หน่วย	LOD	วิธีทดสอบอ้างอิง
4,7,10,13,16,19-Docosahexaenoic acid(C22:6n3)	Not Detected	g/100g	0.01	
Polyunsaturate Fatty acid	Not Detected	g/100g	0.01	
Unsaturated fat	0.04	g/100g	-	
Tran fat	Not Detected	g/100g	0.01	
Omega 3	Not Detected	mg/100g	-	
Omega 6	4.56	mg/100g	-	
Omaga 9	32.92	mg/100g	-	
Total polyphenol (as gallic acid)	0.04	mg/ml	-	Singleton, V. L. and Rossi, J.A.J. (1965) Colorimetry of total polyphenol with phosphomolybdic phosphotungstic acid reagents, Am J. Erol. Vitic., 16: 144-158.
Vitamin C	1.23	mg/100g	-	Compendium of method for food analysis (2003) p2-112 to 2-114
Caffeic Acid	Not Detected	mg/kg	2.00	Analysed by HPLC-DAD/MSD
Flavonoid	< 10.00	mg/kg	-	Colorimetric Method
**Organic acid**				
Citric acid	62.27	mg/kg	-	Analysed by HPLC/DAD
Malic acid	Not Detected	mg/kg	5.00	
Tartaric acid	Not Detected	mg/kg	5.00	
Acetic acid	202.67	mg/kg	-	
Lactic acid	64.13	mg/kg	-	
**Polyphenolic Compound**				
Gallic acid	Not Detected	mg/kg	5.00	In-house method based on Bolivian journal of chemistry
Eriodictyol	Not Detected	mg/kg	5.00	
Apigenin	Not Detected	mg/kg	5.00	
Isoquercetin	Not Detected	mg/kg	5.00	
Kaempferol	Not Detected	mg/kg	5.00	
Quercetin	Not Detected	mg/kg	5.00	

บริษัท ห้องปฏิบัติการกลาง (ประเทศไทย) จำกัด
Central Laboratory (Thailand) Co.,Ltd.
สาขาเชียงใหม่ : 164/86 หมู่ที่ 3 ต.ดอนแก้ว อ.แม่ริม จ.เชียงใหม่ 50180
Chiangmai Branch : 164/86 Moo 3 Donkaew, Maerim, Chiangmai 50180 Thailand
Tel : (66) 0 5389 6131, (66) 0 5389 6133 Fax : (66) 0 5389 6052, (66) 0 5389 6131 ต่อ 705
http://www.centrallabthai.com

Central Lab
One Stop & Fast Services

รายงานผลการทดสอบ

Mature Coconut Water

วันที่ออกรายงาน 25 มิถุนายน 2563
เลขที่รายงาน TRCM63/15996
หน้า 04/04

ผลการทดสอบ

รายการทดสอบ	ผลการทดสอบ	หน่วย	LOD	วิธีทดสอบอ้างอิง
Hydroquinin	Not Detected	mg/kg	5.00	
Rutin	Not Detected	mg/kg	5.00	
Catechin	Not Detected	mg/kg	5.00	
Tannic acid	Not Detected	mg/kg	5.00	

~End of Report~

อนุมัติโดย

(นางสาวศรีวรรณา ฟักน้อย)
ผู้มีอำนาจลงนาม
บริษัท ห้องปฏิบัติการกลาง (ประเทศไทย) จำกัด สาขาเชียงใหม่

รายงานฉบับนี้มีผลเฉพาะกับตัวอย่างที่นำมาทดสอบเท่านั้น
รายงานผลการทดสอบต้องไม่ถูกทำสำเนาเฉพาะเพียงบางส่วน โดยไม่ได้รับความยินยอมเป็นลายลักษณ์อักษรจากห้องปฏิบัติการ ยกเว้นทำทั้งฉบับ
