# Supplementary material for: Novel Coconut Vinegar Attenuates Hepatic and Vascular Oxidative Stress in Rats Fed a High-Cholesterol Diet
Source: Front Nutr. 2022 Mar 9;9:835278. doi: 10.3389/fnut.2022.835278 (PMC8959456; doi:10.3389/fnut.2022.835278)
Supplement: Supplementary file 3 [file Data_Sheet_3.PDF]

Report of Saturated Fatty acid

Batch No.________

Sample received date:

Analyst By: วชิร

Date: 2/6/63

GC code: Coconut Vinegar

Approved By:________

Date:

Sample Code	cm63/04926-001			Duplicate			Average	
Sample Name	น้ำส้มสายชูหมักจากน้ำมะพร้าว			น้ำส้มสายชูหมักจากน้ำมะพร้าว				
Fatty Acid Name	Peak Area	%Area	FA(g/100g	Peak Area	%Area	FA(g/100g)	(g/100g)	
Butyric acid (C4:0)	0.00000	0.00000	0.00000	0.00000	0.00000	0.00000	0.00	ND
Caproic acid (C6:0)	2.49274	27.66655	0.13003	2.49274	27.66655	0.13003	0.13	0.13
Caprylic acid (C8:0)	0.00000	0.00000	0.00000	0.00000	0.00000	0.00000	0.00	ND
Capric acid (C10:0)	0.00000	0.00000	0.00000	0.00000	0.00000	0.00000	0.00	ND
Undecanoic acid (C11:0)	0.00000	0.00000	0.00000	0.00000	0.00000	0.00000	0.00	ND
Lauric acid (C12:0)	0.00000	0.00000	0.00000	0.00000	0.00000	0.00000	0.00	ND
Tridecanoic acid (13:0)	0.00000	0.00000	0.00000	0.00000	0.00000	0.00000	0.00	ND
Myristic acid (C14:0)	2.24943	24.96609	0.11734	2.24943	24.96609	0.11734	0.12	0.12
Pentadecanoic acid(C15:0)	0.00000	0.00000	0.00000	0.00000	0.00000	0.00000	0.00	ND
Palmitic acid (C16:0)	3.40869	37.83255	0.17781	3.40869	37.83255	0.17781	0.18	0.18
Heptadecanoic acid (17:0)	0.00000	0.00000	0.00000	0.00000	0.00000	0.00000	0.00	ND
Stearic acid (C18:0)	0.85908	9.53481	0.04481	0.85908	9.53481	0.04481	0.04	0.04
Arachidic acid (C20:0)	0.00000	0.00000	0.00000	0.00000	0.00000	0.00000	0.00	ND
Heneicosanoic acid(C21:0)	0.00000	0.00000	0.00000	0.00000	0.00000	0.00000	0.00	ND
Behenic acid (C22:0)	0.00000	0.00000	0.00000	0.00000	0.00000	0.00000	0.00	ND
Tricosanoic acid (C23:0)	0.00000	0.00000	0.00000	0.00000	0.00000	0.00000	0.00	ND
Lignoceric acid (C24:0)	0.00000	0.00000	0.00000	0.00000	0.00000	0.00000	0.00	ND
ΣArea	9.0099		ΣArea	9.0099	ΣSaturated fatty acid		0.47	0.47
Total fat	0.47							

PA_{fa} :Peak Area Fatty acid

PA_{in} :Peak Area Internal standard (Undecanoic acid (C11:0))

A_{fa} :%Area Fatty acid

TTF :Total Fat

$$\%Area = \frac{PA_{fa} \times 100}{\sum Area - PA_{in}}$$

$$\text{Fatty Acid (g/100g)} = \frac{A_{fa} \times TTF}{100}$$

(FA)

สำเนาไม่ควบคุม

บริษัท ห้องปฏิบัติการกลาง (ประเทศไทย) จำกัด

สาขาเชียงใหม่

เอกสารฉบับนี้ ขอสงวนสิทธิ์ให้กับลูกค้าบริษัทฯ เท่านั้น

Report of Unsaturated Fatty acid

Batch No.______________

Sample received date:

Analyst By: วชิร

Date: 2/6/63

GC code:______________

Approved By:______________

Date:

Sample Code	cm63/04926-001			Duplicate			Average	
Sample Name	น้ำส้มสายชูหมักจากน้ำมะพร้าว			น้ำส้มสายชูหมักจากน้ำมะพร้าว				
Fatty Acid Name	Peak Area	%Area	FA(g/100g	Peak Area	%Area	FA(g/100g)	(g/100g)	
Myristoleic acid (C14:1)	0.00000	0.00000	0.00000	0.00000	0.00000	0.00000	0.00	ND
cis-10-Pentadecanoic acid(C15:1n10)	0.00000	0.00000	0.00000	0.00000	0.00000	0.00000	0.00	ND
Palmitoleic acid (C16:1n7)	0.00000	0.00000	0.00000	0.00000	0.00000	0.00000	0.00	ND
cis-10-Heptadecanoic acid(C17:1n10)	0.00000	0.00000	0.00000	0.00000	0.00000	0.00000	0.00	ND
trans-9-Elaidic acid(C18:1n9t)	0.00000	0.00000	0.00000	0.00000	0.00000	0.00000	0.00	ND
cis-9-Oleic acid (C18:1n9c)	0.00000	0.00000	0.00000	0.00000	0.00000	0.00000	0.00	ND
cis-11-Eicosenoic acid(C20:1n11)	0.00000	0.00000	0.00000	0.00000	0.00000	0.00000	0.00	ND
Erucic acid (C22:1n9)	0.00000	0.00000	0.00000	0.00000	0.00000	0.00000	0.00	ND
Nervonic acid (C24:1n9)	0.00000	0.00000	0.00000	0.00000	0.00000	0.00000	0.00	ND
			ΣMonounsaturated Fatty acid				0.00	ND
trans-Linolelaidic acid(C18:2n6t)	0.00000	0.00000	0.00000	0.00000	0.00000	0.00000	0.00	ND
cis-9,12-Linoleic acid (C18:2n6)	0.00000	0.00000	0.00000	0.00000	0.00000	0.00000	0.00	ND
Gamma-Linolenic acid (C18:3n6)	0.00000	0.00000	0.00000	0.00000	0.00000	0.00000	0.00	ND
Alpha-Linolenic acid (C18:3n3)	0.00000	0.00000	0.00000	0.00000	0.00000	0.00000	0.00	ND
cis-11,14-Eicosadienoic acid (C20:2)	0.00000	0.00000	0.00000	0.00000	0.00000	0.00000	0.00	ND
cis-8,11,14-Eicosatrienoic acid (C20:3n6)	0.00000	0.00000	0.00000	0.00000	0.00000	0.00000	0.00	ND
cis-11,14,17-Eicosatrienoic acid (C20:3n3)	0.00000	0.00000	0.00000	0.00000	0.00000	0.00000	0.00	ND
Arachidonic acid (C20:4n6)	0.00000	0.00000	0.00000	0.00000	0.00000	0.00000	0.00	ND
cis-13,16-Docosadienoic acid(C22:2)	0.00000	0.00000	0.00000	0.00000	0.00000	0.00000	0.00	ND
cis-5,8,11,14,17-Eicosapentaenoic acid(C20:5n3)	0.00000	0.00000	0.00000	0.00000	0.00000	0.00000	0.00	ND
cis-4,7,10,13,16,19-Docosahexaenoic acid(C22:6n3)	0.00000	0.00000	0.00000	0.00000	0.00000	0.00000	0.00	ND
ΣArea	9.0099			ΣArea	9.0099			
Total fat	0.47							

ΣPolyunsaturated fatty acid	0.00	ND
ΣUnsaturated Fatty acid	0.00	ND
Trans Fatty acid	0.00	ND

OMEGA 3	0.00	mg/100g
OMEGA6	0.00	mg/100g
OMEGA9	0.00	mg/100g

สำเนาไม่ควบคุม

บริษัท ห้องปฏิบัติการกลาง (ประเทศไทย) จำกัด

สาขาเชียงใหม่

เอกสารฉบับนี้ ขอสงวนสิทธิ์ให้กับลูกค้าบริษัทฯ เท่านั้น

Injection Date	: 6/2/2020 8:00:36 PM	Seq. Line :	4
Sample Name	: cm63/4926-001	Location :	Vial 3
Acq. Operator	: wachira	Inj :	1
Acq. Instrument	: Instrument 1	Inj Volume :	1 µl
Acq. Method	: C:\HPCHEM\1\METHODS\FFA.M		
Last changed	: 6/2/2020 3:34:12 PM by wachira		
Analysis Method	: C:\HPCHEM\1\METHODS\ST051263.M		
Last changed	: 5/14/2020 4:48:59 PM by wachira		

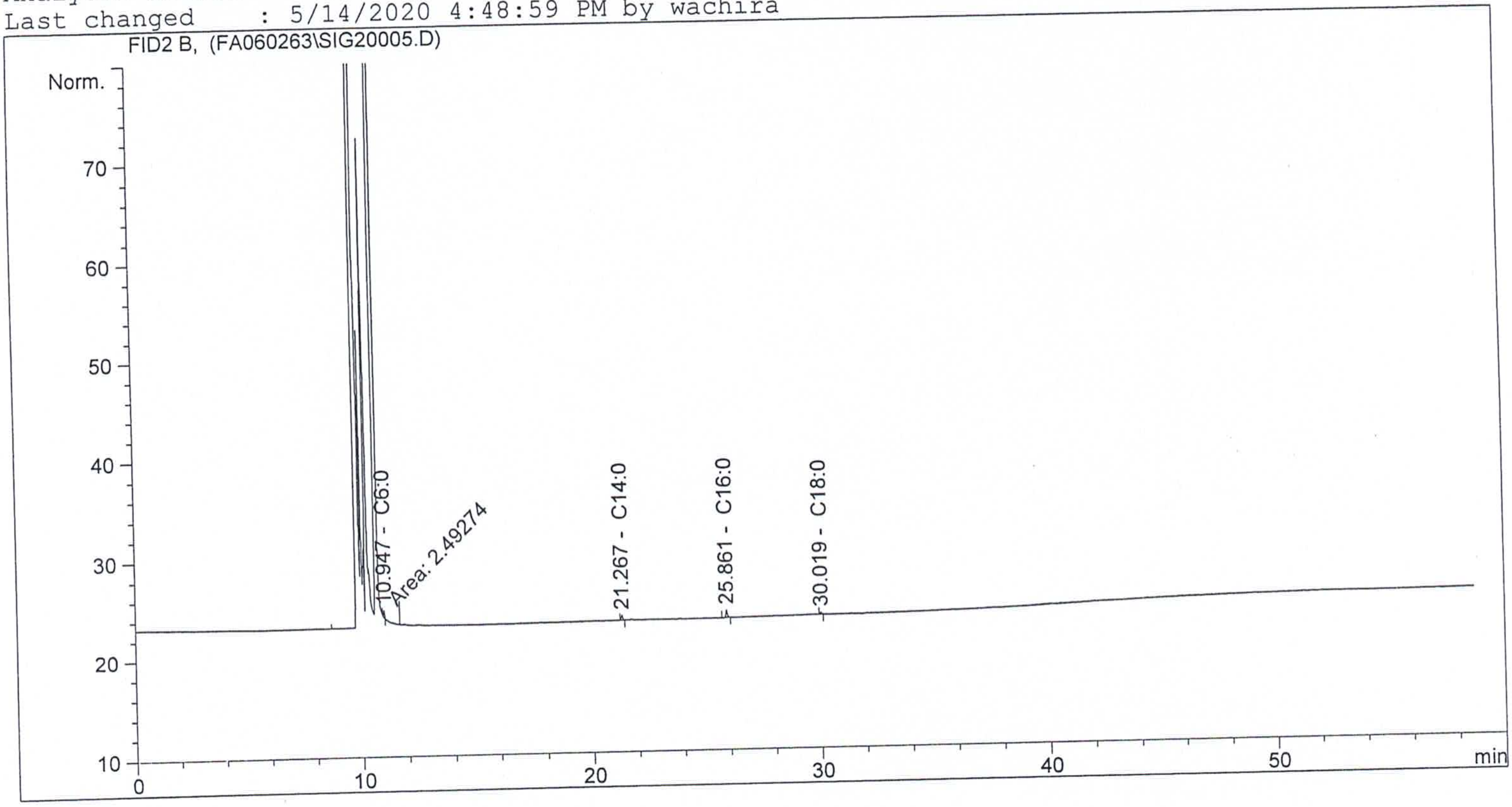

Area Percent Report

Sorted By	:	Signal
Calib. Data Modified	:	4/30/2020 2:29:42 PM
Multiplier	:	1.0000
Dilution	:	1.0000

Use Multiplier & Dilution Factor with ISTDs

Signal 1: FID2 B,

Peak #	RetTime [min]	Type	Width [min]	Area [pA*s]	Area %	Name
1	10.448		0.0000	0.00000	0.00000	C4:0
2	10.947	MM	0.0568	2.49274	27.66652	C6:0
3	12.447		0.0000	0.00000	0.00000	C8:0
4	14.607		0.0000	0.00000	0.00000	C10:0
5	16.080		0.0000	0.00000	0.00000	C11:0
6	17.777		0.0000	0.00000	0.00000	C12:0
7	19.689		0.0000	0.00000	0.00000	C13:0
8	21.267	BB	0.0631	2.24943	24.96613	C14:0
9	23.542		0.0000	0.00000	0.00000	C14:1
10	23.819		0.0000	0.00000	0.00000	C15:0
11	25.672		0.0000	0.00000	0.00000	C15:1
12	25.861	BB	0.0649	3.40869	37.83253	C16:0
13	27.497		0.0000	0.00000	0.00000	C16:1
14	28.029		0.0000	0.00000	0.00000	C17:0

สำเนาไม่ควบคุม
บริษัท ห้องปฏิบัติการกลาง (ประเทศไทย) จำกัด
สาขาเชียงใหม่
เอกสารฉบับนี้ ขอสงวนสิทธิ์ให้กับลูกค้าบริษัทฯ [illegible]

Peak #	RetTime [min]	Type	Width [min]	Area [pA*s]	Area %	Name
15	29.566		0.0000	0.00000	0.00000	C17:1
16	30.019	BP	0.0513	8.59081e-1	9.53482	C18:0
17	31.002		0.0000	0.00000	0.00000	C18:1n9t
18	31.399		0.0000	0.00000	0.00000	C18:1n9c
19	32.462		0.0000	0.00000	0.00000	C18:2n6t
20	33.357		0.0000	0.00000	0.00000	C18:2n6c
21	33.971		0.0000	0.00000	0.00000	C20:0
22	34.780		0.0000	0.00000	0.00000	C18:3n6 (gamma)
23	35.234		0.0000	0.00000	0.00000	C20:1n11c
24	35.550		0.0000	0.00000	0.00000	C18:3n3 (alpha)
25	35.833		0.0000	0.00000	0.00000	C21:0
26	37.089		0.0000	0.00000	0.00000	C20:2
27	37.621		0.0000	0.00000	0.00000	C22:0
28	38.440		0.0000	0.00000	0.00000	C20:3n6
29	38.822		0.0000	0.00000	0.00000	C22:1n9
30	39.151		0.0000	0.00000	0.00000	C20:3n3
31	39.356		0.0000	0.00000	0.00000	C23:0
32	39.471		0.0000	0.00000	0.00000	C20:4n6
33	40.570		0.0000	0.00000	0.00000	C22:2
34	41.032		0.0000	0.00000	0.00000	C24:0
35	41.588		0.0000	0.00000	0.00000	C20:5n3 (EPA)
36	42.177		0.0000	0.00000	0.00000	C24:1n9
37	46.266		0.0000	0.00000	0.00000	C22:6n3 (DHA)
Totals :				9.00994		

Results obtained with enhanced integrator!
2 Warnings or Errors :

Warning : Calibration warnings (see calibration table listing)
Warning : Calibrated compound(s) not found

==

*** End of Report ***

สำเนาไม่ควบคุม
บริษัท ห้องปฏิบัติการกลาง (ประเทศไทย) จำกัด
สาขาเชียงใหม่
เอกสารฉบับนี้ ขอสงวนสิทธิ์ให้กับลูกค้าบริษัทฯ เท่านั้น

Report of Saturated Fatty acid

Batch No.__________

Sample received date:

Analyst By: วชิร

Date: 2/6/63

GC code: Mature Coconut Water

Approved By:__________

Date:

Sample Code	cm63/04926-002			Duplicate			Average	
Sample Name	น้ำมะพร้าวแกง			น้ำมะพร้าวแกง				
Fatty Acid Name	Peak Area	%Area	FA(g/100g	Peak Area	%Area	FA(g/100g)	(g/100g)	
Butyric acid (C4:0)	0.00000	0.00000	0.00000	0.00000	0.00000	0.00000	0.00	ND
Caproic acid (C6:0)	1.46131	0.39323	0.00134	1.46131	0.39323	0.00134	0.00	ND
Caprylic acid (C8:0)	19.02416	5.11924	0.01741	19.02416	5.11924	0.01741	0.02	0.02
Capric acid (C10:0)	14.72243	3.96168	0.01347	14.72243	3.96168	0.01347	0.01	0.01
Undecanoic acid (C11:0)	0.00000	0.00000	0.00000	0.00000	0.00000	0.00000	0.00	ND
Lauric acid (C12:0)	146.59877	39.44850	0.13412	146.59877	39.44850	0.13412	0.13	0.13
Tridecanoic acid (13:0)	0.00000	0.00000	0.00000	0.00000	0.00000	0.00000	0.00	ND
Myristic acid (C14:0)	78.10680	21.01789	0.07146	78.10680	21.01789	0.07146	0.07	0.07
Pentadecanoic acid(C15:0)	0.00000	0.00000	0.00000	0.00000	0.00000	0.00000	0.00	ND
Palmitic acid (C16:0)	50.94348	13.70846	0.04661	50.94348	13.70846	0.04661	0.05	0.05
Heptadecanoic acid (17:0)	0.00000	0.00000	0.00000	0.00000	0.00000	0.00000	0.00	ND
Stearic acid (C18:0)	17.12250	4.60752	0.01567	17.12250	4.60752	0.01567	0.02	0.02
Arachidic acid (C20:0)	0.80052	0.21541	0.00073	0.80052	0.21541	0.00073	0.00	ND
Heneicosanoic acid(C21:0)	0.00000	0.00000	0.00000	0.00000	0.00000	0.00000	0.00	ND
Behenic acid (C22:0)	0.80352	0.21622	0.00074	0.80352	0.21622	0.00074	0.00	ND
Tricosanoic acid (C23:0)	0.00000	0.00000	0.00000	0.00000	0.00000	0.00000	0.00	ND
Lignoceric acid (C24:0)	1.06928	0.28773	0.00098	1.06928	0.28773	0.00098	0.00	ND
$\sum$Area	371.6206			$\sum$Area	371.6206	$\sum$Saturated fatty acid	0.30	0.30
Total fat	0.34							

$$\%\text{Area} = \frac{PA_{fa} \times 100}{\sum \text{Area} - PA_{in}}$$

$$\text{Fatty Acid (g/100g)} = \frac{A_{fa} \times TTF}{100}$$

(FA)

PA_{fa} :Peak Area Fatty acid

PA_{in} :Peak Area Internal standard (Undecanoic acid (C11:0))

A_{fa} :%Area Fatty acid

TTF :Total Fat

สำเนาไม่ควบคุม

บริษัท ห้องปฏิบัติการกลาง (ประเทศไทย) จำกัด

สาขาเชียงใหม่

เอกสารฉบับนี้ ขอสงวนสิทธิ์ให้กับลูกค้าบริษัทฯ เท่านั้น

Report of Unsaturated Fatty acid — Batch No.______________

Sample received date: — Analyst By: วชิร — Date: 2/6/63

GC code:______________ — Approved By:______________ — Date:

Sample Code	cm63/04926-002			Duplicate			Average	
Sample Name	น้ำมะพร้าวแกง			น้ำมะพร้าวแกง				
Fatty Acid Name	Peak Area	%Area	FA(g/100g	Peak Area	%Area	FA(g/100g)	(g/100g)	
Myristoleic acid (C14:1)	0.00000	0.00000	0.00000	0.00000	0.00000	0.00000	0.00	ND
cis-10-Pentadecanoic acid(C15:1n10)	0.00000	0.00000	0.00000	0.00000	0.00000	0.00000	0.00	ND
Palmitoleic acid (C16:1n7)	0.00000	0.00000	0.00000	0.00000	0.00000	0.00000	0.00	ND
cis-10-Heptadecanoic acid(C17:1n10)	0.00000	0.00000	0.00000	0.00000	0.00000	0.00000	0.00	ND
trans-9-Eladic acid(C18:1n9t)	0.00000	0.00000	0.00000	0.00000	0.00000	0.00000	0.00	ND
cis-9-Oleic acid (C18:1n9c)	35.98233	9.68254	0.03292	35.98233	9.68254	0.03292	0.03	0.03
cis-11-Eicosenoic acid(C20:1n11)	0.00000	0.00000	0.00000	0.00000	0.00000	0.00000	0.00	ND
Erucic acid (C22:1n9)	0.00000	0.00000	0.00000	0.00000	0.00000	0.00000	0.00	ND
Nervonic acid (C24:1n9)	0.00000	0.00000	0.00000	0.00000	0.00000	0.00000	0.00	ND
			ΣMonounsaturated Fatty acid				0.03	0.03
trans-Linolelaidic acid(C18:2n6t)	0.00000	0.00000	0.00000	0.00000	0.00000	0.00000	0.00	ND
cis-9,12-Linoleic acid (C18:2n6)	4.98553	1.34156	0.00456	4.98553	1.34156	0.00456	0.00	ND
Gamma-Linolenic acid (C18:3n6)	0.00000	0.00000	0.00000	0.00000	0.00000	0.00000	0.00	ND
Alpha-Linolenic acid (C18:3n3)	0.00000	0.00000	0.00000	0.00000	0.00000	0.00000	0.00	ND
cis-11,14-Eicosadienoic acid (C20:2)	0.00000	0.00000	0.00000	0.00000	0.00000	0.00000	0.00	ND
cis-8,11,14-Eicosatrienoic acid (C20:3n6)	0.00000	0.00000	0.00000	0.00000	0.00000	0.00000	0.00	ND
cis-11,14,17-Eicosatrienoic acid (C20:3n3)	0.00000	0.00000	0.00000	0.00000	0.00000	0.00000	0.00	ND
Arachidonic acid (C20:4n6)	0.00000	0.00000	0.00000	0.00000	0.00000	0.00000	0.00	ND
cis-13,16-Docosadienoic acid(C22:2)	0.00000	0.00000	0.00000	0.00000	0.00000	0.00000	0.00	ND
cis-5,8,11,14,17-Eicosapentaenoic acid(C20:5n3)	0.00000	0.00000	0.00000	0.00000	0.00000	0.00000	0.00	ND
cis-4,7,10,13,16,19-Docosahexaenoic acid(C22:6n3)	0.00000	0.00000	0.00000	0.00000	0.00000	0.00000	0.00	ND
ΣArea	371.6206		ΣArea	371.6206				
Total fat	0.34		ΣPolyunsaturated fatty acid				0.00	ND
			ΣUnsaturated Fatty acid				0.04	0.04
					Trans Fatty acid		0.00	ND

OMEGA 3	0.00	mg/100g
OMEGA6	4.56	mg/100g
OMEGA9	32.92	mg/100g

สำเนาไม่ควบคุม
บริษัท ห้องปฏิบัติการกลาง (ประเทศไทย) จำกัด
สาขาเชียงใหม่
เอกสารฉบับนี้ ขอสงวนลิขสิทธิ์ให้กับลูกค้าบริษัทฯ เท่านั้น

===

Injection Date : 6/2/2020 9:07:22 PM
Sample Name : cm63/4926-002
Acq. Operator : wachira
Acq. Instrument : Instrument 1
Acq. Method : C:\HPCHEM\1\METHODS\FFA.M
Last changed : 6/2/2020 3:34:12 PM by wachira
Analysis Method : C:\HPCHEM\1\METHODS\ST051263.M
Last changed : 5/14/2020 4:48:59 PM by wachira

Seq. Line : 5
Location : Vial 4
Inj : 1
Inj Volume : 1 µl

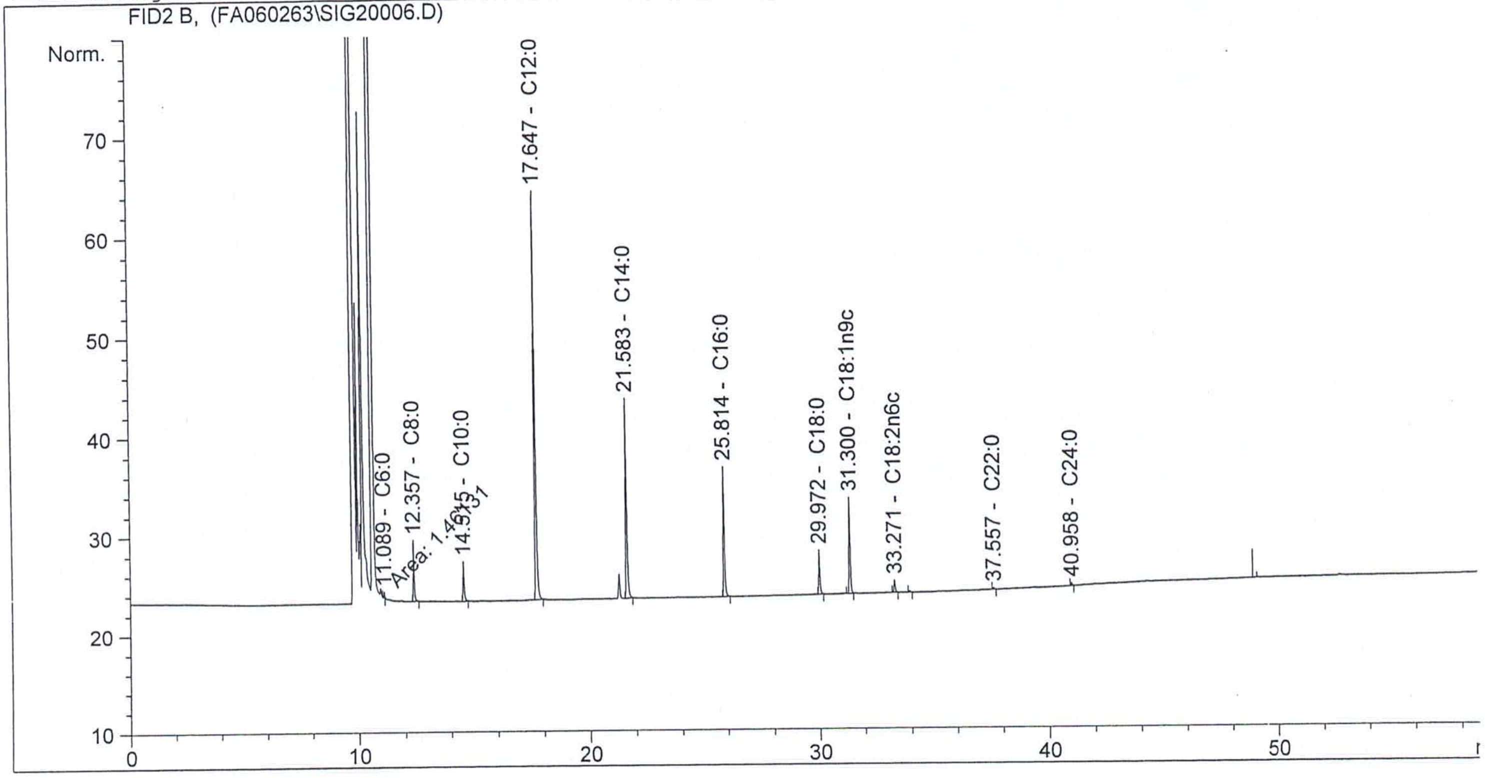

===

Area Percent Report

===

Sorted By : Signal
Calib. Data Modified : 4/30/2020 2:29:42 PM
Multiplier : 1.0000
Dilution : 1.0000
Use Multiplier & Dilution Factor with ISTDs

Signal 1: FID2 B,

Peak #	RetTime [min]	Type	Width [min]	Area [pA*s]	Area %	Name
1	10.448		0.0000	0.00000	0.00000	C4:0
2	11.089	MM	0.0431	1.46131	0.39323	C6:0
3	12.357	VB	0.0470	19.02416	5.11924	C8:0
4	14.515	BB	0.0537	14.72243	3.96168	C10:0
5	16.080		0.0000	0.00000	0.00000	C11:0
6	17.647	BB	0.0538	146.59877	39.44850	C12:0
7	19.689		0.0000	0.00000	0.00000	C13:0
8	21.583	VB	0.0583	78.10680	21.01789	C14:0
9	23.542		0.0000	0.00000	0.00000	C14:1
10	23.819		0.0000	0.00000	0.00000	C15:0
11	25.672		0.0000	0.00000	0.00000	C15:1
12	25.814	BP	0.0585	50.94348	13.70846	C16:0
13	27.497		0.0000	0.00000	0.00000	C16:1
14	28.029		0.0000	0.00000	0.00000	C17:0

สำเนาไม่ควบคุม
บริษัท ห้องปฏิบัติการกลาง (ประเทศไทย) จำกัด
สาขาเชียงใหม่
เอกสารฉบับนี้ ขอสงวนสิทธิ์ให้กับลูกค้าบริษัทฯ เท่านั้น

Peak #	RetTime [min]	Type	Width [min]	Area [pA*s]	Area %	Name
15	29.566		0.0000	0.00000	0.00000	C17:1
16	29.972	BB	0.0581	17.12250	4.60752	C18:0
17	31.002		0.0000	0.00000	0.00000	C18:1n9t
18	31.300	PB	0.0575	35.98233	9.68254	C18:1n9c
19	32.462		0.0000	0.00000	0.00000	C18:2n6t
20	33.271	PP	0.0581	4.98553	1.34156	C18:2n6c
21	33.917	BP	0.0553	8.00518e-1	0.21541	C20:0
22	34.780		0.0000	0.00000	0.00000	C18:3n6 (gamma)
23	35.234		0.0000	0.00000	0.00000	C20:1n11c
24	35.550		0.0000	0.00000	0.00000	C18:3n3 (alpha)
25	35.833		0.0000	0.00000	0.00000	C21:0
26	37.089		0.0000	0.00000	0.00000	C20:2
27	37.557	BP	0.0509	8.03516e-1	0.21622	C22:0
28	38.440		0.0000	0.00000	0.00000	C20:3n6
29	38.822		0.0000	0.00000	0.00000	C22:1n9
30	39.151		0.0000	0.00000	0.00000	C20:3n3
31	39.356		0.0000	0.00000	0.00000	C23:0
32	39.471		0.0000	0.00000	0.00000	C20:4n6
33	40.570		0.0000	0.00000	0.00000	C22:2
34	40.958	BP	0.0513	1.06928	0.28773	C24:0
35	41.588		0.0000	0.00000	0.00000	C20:5n3 (EPA)
36	42.177		0.0000	0.00000	0.00000	C24:1n9
37	46.266		0.0000	0.00000	0.00000	C22:6n3 (DHA)
Totals :				371.62062		

Results obtained with enhanced integrator!
2 Warnings or Errors :

Warning : Calibration warnings (see calibration table listing)
Warning : Calibrated compound(s) not found

*** End of Report ***

สำเนาไม่ควบคุม
บริษัท ห้องปฏิบัติการกลาง (ประเทศไทย) จำกัด
สาขาเชียงใหม่
เอกสารฉบับนี้ ขอสงวนสิทธิ์ให้กับลูกค้าบริษัทฯ เท่านั้น
