# Supplementary material for: Novel Coconut Vinegar Attenuates Hepatic and Vascular Oxidative Stress in Rats Fed a High-Cholesterol Diet
Source: Front Nutr. 2022 Mar 9;9:835278. doi: 10.3389/fnut.2022.835278 (PMC8959456; doi:10.3389/fnut.2022.835278)
Supplement: Supplementary file 4 [file Data_Sheet_4.PDF]

ห้องปฏิบัติการกลางตรวจสอบผลิตภัณฑ์เกษตรและอาหาร จำกัด
เลขที่ 164/86 หมู่ 3 ตำบล ดอนแก้ว อำเภอ แม่ริม จังหวัด เชียงใหม่ 50180 Tel:05-3896131 Fax:05-3896052

Total polyphenol Analysis Worksheet

Sample name: น้ำส้มสายชู Coconut Vinegar Sample Code: CM63/0A926-001

Sample received date: ______

Item	I	II	Calibration curve gallic acid
Wt. of sample (g) ml	10	10	ค่าความเข้มข้นที่ 10 -250 ppm
ค่า Absorbance ที่ 765 nm	0.15845	0.16228	R^2=...............................
Amount (μg/mL)	32.267	33.04800	Y = mx +c.......................
Total polyphenol (mg/g)	0.16133	0.16524	
Average total polyphenol (mg/g)	0.16		

Total polyhpenol (mg/g) wet basis = $\frac{\text{Amount ที่ได้จากกราฟ } (\mu g/mL) \times \text{Dilution facterที่ใช้} \times \text{250 (50)}}{\text{(10) Wt. of sample (g)} \times 1000}$

Total polyhpenol (mg/g) dry basis = $\frac{\text{Amount ที่ได้จากกราฟ } (\mu g/mL) \times \text{Dilution facterที่ใช้} \times 250 \times 100}{(100-\%M) \times \text{Wt. of sample (g)} \times 1000}$

Tested by: [signature] Approved by: [signature]

Date: 2/6/63 Date: 2/6/63

Reference Method : Singleton, V.L., Rossi, J.A.J. (1965) Colorimetry of total phenolics with phosphomolybdic – Phosphotungstic acid reagents, Am.J.Erol. Vitic, 16:144-158

ผู้อนุมัติ .... 25/6/6[illegible]
ผู้จำหน่าย .... 25/6/6[illegible]

ห้องปฏิบัติการกลางตรวจสอบผลิตภัณฑ์เกษตรและอาหาร จำกัด
เลขที่ 164/86 หมู่ 3 ตำบล ดอนแก้ว อำเภอ แม่ริม จังหวัด เชียงใหม่ 50180 Tel:05-3896131 Fax:05-3896052

สำเนาไม่ควบคุม

Total polyphenol Analysis Worksheet

บริษัท ห้องปฏิบัติการกลาง (ประเทศไทย) จำกัด
สาขาเชียงใหม่
เอกสารฉบับนี้ ขอสงวนสิทธิ์ใช้กับลูกค้าบริษัทฯ เท่า[illegible]

Sample name: น้ำมะพร้าวแก่ Mature Coconut water Sample Code: CM63[illegible]002

Sample received date: ______

Item	I	II	Calibration curve gallic acid
Wt. of sample (g) ml	10	10	ค่าความเข้มข้นที่ 10 -250 ppm
ค่า Absorbance ที่ 765 nm	0.040236	0.041177	R^2=...............................
Amount (μg/mL)	8.19400	8.38560	Y = mx +c.......................
Total polyphenol (mg/g)	0.04097	0.041928	
Average total polyphenol (mg/g)	0.04		

Total polyhpenol (mg/g) wet basis = $\frac{\text{Amount ที่ได้จากกราฟ } (\mu g/mL) \times \text{Dilution facterที่ใช้} \times \text{250 (50)}}{\text{(10) Wt. of sample (g)} \times 1000}$

Total polyhpenol (mg/g) dry basis = $\frac{\text{Amount ที่ได้จากกราฟ } (\mu g/mL) \times \text{Dilution facterที่ใช้} \times 250 \times 100}{(100-\%M) \times \text{Wt. of sample (g)} \times 1000}$

Tested by: [signature] Approved by: [signature]

Date: 2/6/63 Date: 2/6/63

Reference Method : Singleton, V.L., Rossi, J.A.J. (1965) Colorimetry of total phenolics with phosphomolybdic – Phosphotungstic acid reagents, Am.J.Erol. Vitic, 16:144-158

Last Blank Spectrum

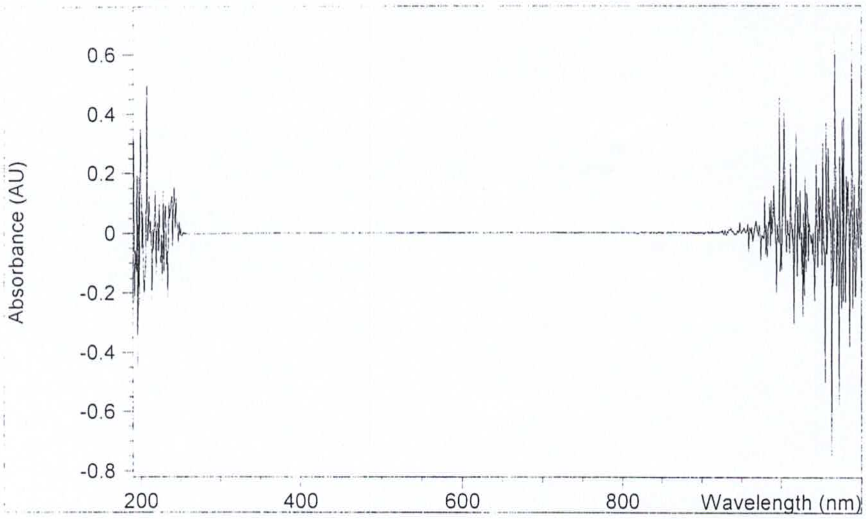

Processed Standard Spectra

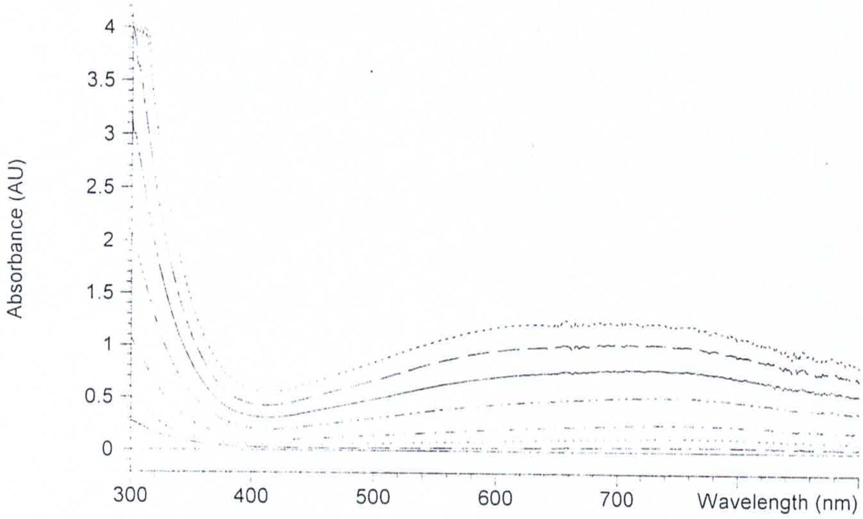

Calibration Curve

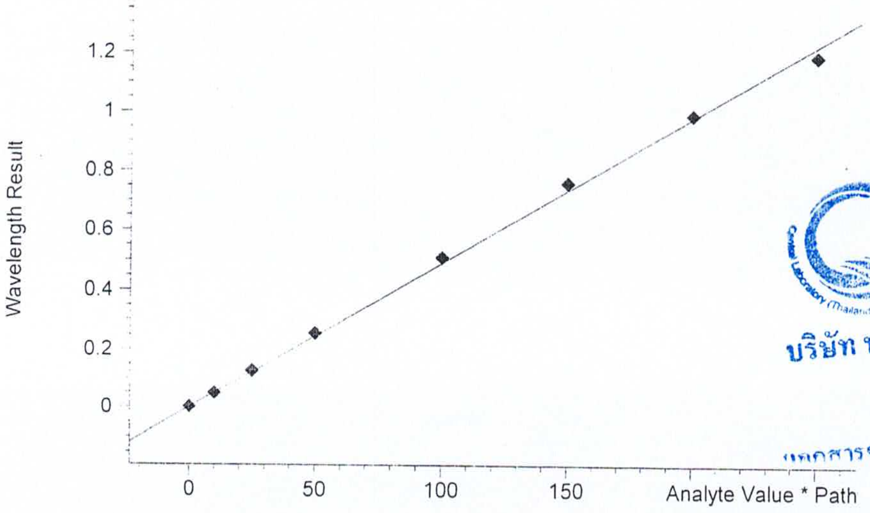

สำเนาไม่ควบคุม
บริษัท ห้องปฏิบัติการกลาง (ประเทศไทย) จำกัด
สาขาเชียงใหม่
เอกสารฉบับนี้ ขอสงวนสิทธิ์ให้กับลูกค้าบริษัทฯ เท่านั้น

Calibration Table

#	Standard Name	Concentration(ug/ml)	Abs<765nm>	%Error
1	std 0	0.00000	6.8521E-4	-100.00
2	std 10	10.00000	4.8611E-2	1.02
3	std 25	25.00000	0.12930	-5.06
4	std 50	50.00000	0.25642	-4.25
5	std 100	100.00000	0.51077	-3.86
6	std 150	150.00000	0.76114	-3.23
7	std 200	200.00000	0.99193	-0.99
8	std 250	250.00000	1.19230	2.97

Calibration Result Summary

Analyte Name	Concentration	Std.Dev. of k1	2.10960 ug/ml
Number of Standards	8	Std.Dev. of Calibrat	3.84990 ug/ml
Calibration Curve	C = k1 * A	Correl. Coeff. (R^2)	0.99925
Coefficient k1	203.65000 ug/ml		

*** End Hardcopy view ***

สำเนาไม่ควบคุม
บริษัท ห้องปฏิบัติการกลาง (ประเทศไทย) จำกัด
สาขาเชียงใหม่
เอกสารฉบับนี้ ขอสงวนสิทธิ์ให้กับลูกค้าบริษัทฯ เท่านั้น

Last Blank Spectrum

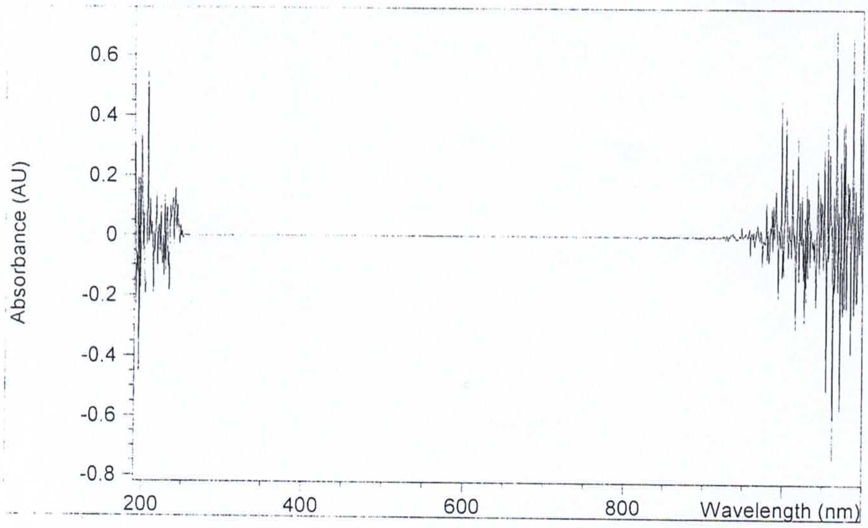

Overlaid Sample Spectra

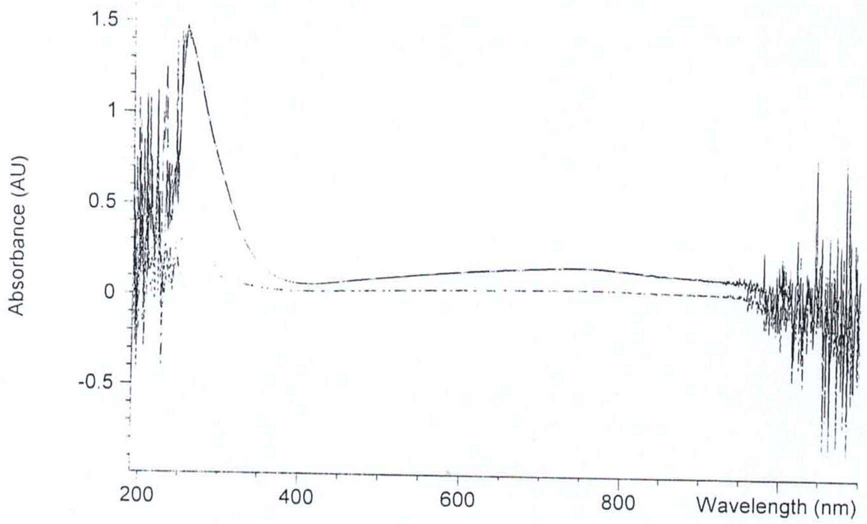

Sample/Result Table

#	Name	Dilut. Factor	Concentration(ug/ml)	Abs<765nm>
1	cm63/4926-001/1	1.00000	32.26700	0.15845
2	cm63/4926-001/2	1.00000	33.04800	0.16228
3	cm63/4926-002/1	1.00000	8.19400	4.0236E-2
4	cm63/4926-002/2	1.00000	8.38560	4.1177E-2

Calibration Result Summary

Analyte Name	Concentration
Number of Standards	8
Calibration Curve	C = k1 * A
Coefficient k1	203.65000 ug/ml

Std.Dev. of k1	2.10960 ug/ml
Std.Dev. of Calibrat	3.84990 ug/ml
Correl. Coeff. (R^2)	0.99925

สำเนาไม่ควบคุม

บริษัท ห้องปฏิบัติการกลาง (ประเทศไทย) จำกัด

สาขาเชียงใหม่

เอกสารฉบับนี้ ขอสงวนสิทธิ์ให้กับลูกค้าบริษัทฯ เท่านั้น
