# Supplementary material for: Novel Coconut Vinegar Attenuates Hepatic and Vascular Oxidative Stress in Rats Fed a High-Cholesterol Diet
Source: Front Nutr. 2022 Mar 9;9:835278. doi: 10.3389/fnut.2022.835278 (PMC8959456; doi:10.3389/fnut.2022.835278)
Supplement: Supplementary file 5 [file Data_Sheet_5.PDF]

# Vitamin C Analysis Worksheet

วันที่รับตัวอย่าง 2/6/63
วันที่ทดสอบ 2/6/63
Balance No. BL2-02-CM

| chemical | | |
|---|---|---|
| Chemical names | Lot No. | Purity |
| L-ascorbate | | |
| $KH_2PO_4$ | | |
| Methanol | | |

| system suitability | | |
|---|---|---|
| parameter | Criterion | Results |
| 1. number of plate (n) | >2000 | |
| 2. peak symmetry | ≤ 2 | |
| 3. Tailing | ≤ 2 | |
| 4. Repeatabity of injection (%RSD) | < 2 % | |

**Working solution of Vitamin C**

Stock Vitamin C ..... 1 ..... mg/mL

**Injection of Vitamin C**

HPLC No. LCD-03-CM

Inject by [signature]

สูตรคำนวณ Vitamin C (mg/100g) = $\frac{C \times 50 \times 100}{\text{Wt.sample (g)}}$

sodium ascorbate = 1.12

potassium ascorbate = 1.22

Control samples

| Sample Code | Sample name | Wt.sample(g) | Vitamin C (mg/ml) | Vitamin C (mg/100g) | Spike conc. (mg/100g) | % Recovery |
|---|---|---|---|---|---|---|
| 02867-004-3 | เจลเกลือแร่ | 5.00 | 0.154084 | 154.0840 | 0.0500 | 104.03 |
| | | | | | | |

Samples

| Sample code | Sample name | Wt.sample(g) | Vitamin C (mg/ml) | Vitamin C (mg/100g) | average (mg/100g) | % RPD |
|---|---|---|---|---|---|---|
| 04657-001 | Delarm Org. Kale Powder | 5.00 | 0.030829 | 30.8285 | = 30.83 | |
| 04713-001 | มะขามผง | 5.00 | 0.000394 | 0.3939 | = 0.39 | |
| 04800-001-1 | gummy jelly | 5.00 | 0.101560 | 101.5600 | 102.07 | -1.00 |
| 04800-001-2 | gummy jelly | 5.00 | 0.102581 | 102.5810 | | |
| 04926-001 | น้ำส้มสายชูหมักจากมะพร้าว | 5.00 | 0.000416 | 0.4163 | = 0.42 | Coconut Vinegar |
| 04926-002 | น้ำมะพร้าวแก่ | 5.00 | 0.001228 | 1.2279 | = 1.23 | Mature Coconut Water |
| | | | | | | |
| | | | | | | |
| | | | | | | |
| | | | | | | |
| | | | | | | |
| | | | | | | |
| | | | | | | |
| | | | | | | |
| | | | | | | |
| | | | | | | |
| | | | | | | |

สำเนาไม่ควบคุม
บริษัท ห้องปฏิบัติการกลาง (ประเทศไทย) จำกัด
สาขาเชียงใหม่
เอกสารฉบับนี้ ขอสงวนสิทธิ์ให้กับลูกค้าบริษัทฯ เท่านั้น

| | |
|---|---|
| LOD(mg/100g) | |
| LOQ(mg/100g) | |

Analyzed by [signature] Date 5/6/63
Approved by [signature] Date 5/6/63

Reference Method: In house method base on Food control 16 (2005) 273-277

## Calibration Table

```
Calib. Data Modified      :      4/6/2020 15:26:21 PM

Calculate                 :      External Standard
Based on                  :      Peak Area

Rel. Reference Window :      5.000 %
Abs. Reference Window :      0.000 min
Rel. Non-ref. Window  :      5.000 %
Abs. Non-ref. Window  :      0.000 min
Multiplier                :      1.0000
Dilution                  :      1.0000
Sample Amount             :       0.00000
Use Multiplier & Dilution Factor with ISTDs
Uncalibrated Peaks        :      not reported
Partial Calibration       :      Yes, identified peaks are recalibrated
Correct All Ret. Times:      No, only for identified peaks

Curve Type                :      Linear
Origin                    :      Included
Weight                    :      Equal

Recalibration Settings:
Average Response          :      Average all calibrations
Average Retention Time:      Floating Average New 75%

Calibration Report Options :
    Printout of recalibrations within a sequence:
        Calibration Table after Recalibration
        Normal Report after Recalibration
    If the sequence is done with bracketing:
        Results of first cycle (ending previous bracket)
```

Signal 1: DAD1 A, Sig=244,8 Ref=off

| RetTime [min] | Lvl Sig | | Amount [g/L] | Area | Amt/Area | Ref | Grp | Name |
|---|---|---|---|---|---|---|---|---|
| 3.143 | 1 | 1 | 1.00000e-3 | 38.89258 | 2.57118e-5 | | | Vitamin C |
| | | 2 | 5.00000e-3 | 254.34193 | 1.96586e-5 | | | |
| | | 3 | 1.00000e-2 | 531.21008 | 1.88249e-5 | | | |
| | | 4 | 5.00000e-2 | 2776.81543 | 1.80062e-5 | | | |
| | | 5 | 1.00000e-1 | 5599.77344 | 1.78579e-5 | | | |

## Peak Sum Table

***No Entries in table***

## Calibration Curves

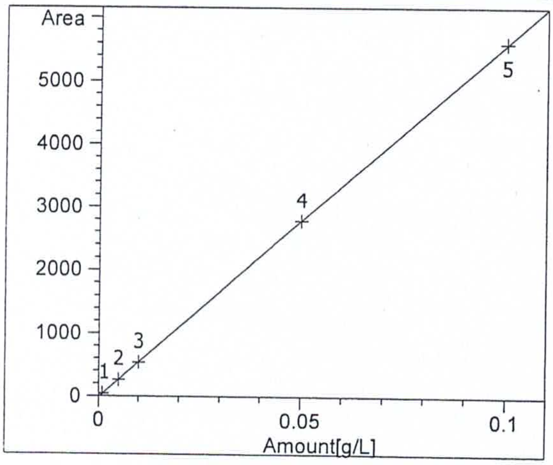


```
Vitamin C at exp. RT: 3.143
DAD1 A, Sig=244,8 Ref=off
Correlation:              0.99999
Residual Std. Dev.:    13.10328
Formula: y = mx + b
        m:   56126.00322
        b:      -19.31385
        x: Amount[g/L]
        y: Area
```

สำเนาไม่ควบคุม
บริษัท ห้องปฏิบัติการกลาง (ประเทศไทย) จำกัด
สาขาเชียงใหม่
เอกสารฉบับนี้ ขอสงวนสิทธิ์ให้กับลูกค้าบริษัทฯ เท่านั้น

```
=====================================================================
Injection Date    : 2/6/2020 18:12:00 PM            Seq. Line :    2
Sample Name       : reagent blank                    Location : Vial 1
Acq. Operator     : supansa                               Inj :    1
Acq. Instrument   : Instrument 1                   Inj Volume : 20 µl
Acq. Method       : C:\HPCHEM\1\METHODS\VIT_C.M
Last changed      : 18/11/2019 15:08:57 PM by supansa
Analysis Method   : C:\HPCHEM\1\METHODS\VC060263.M
Last changed      : 4/6/2020 15:26:22 PM by supansa
```

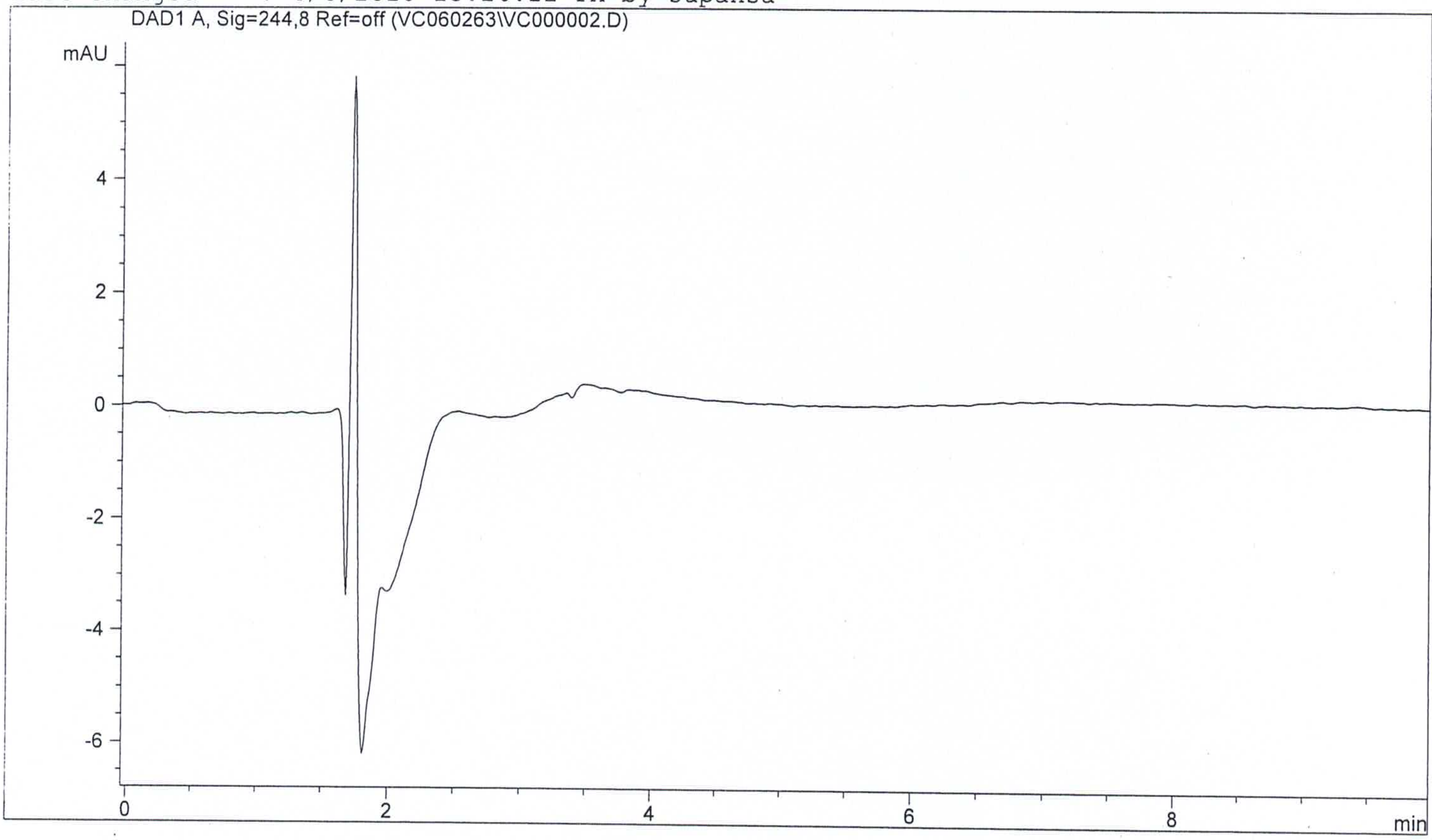


```
=====================================================================
                    External Standard Report
=====================================================================

Sorted By             :      Signal
Calib. Data Modified  :      4/6/2020 15:26:21 PM
Multiplier            :      1.0000
Dilution              :      1.0000
Use Multiplier & Dilution Factor with ISTDs

Signal 1: DAD1 A, Sig=244,8 Ref=off

RetTime  Type    Area      Amt/Area    Amount    Grp   Name
 [min]         [mAU*s]                 [g/L]
-------|------|----------|----------|----------|--|------------------
  3.143             -          -          -          Vitamin C

Totals :                                0.00000

 Results obtained with enhanced integrator!
1 Warnings or Errors :

Warning : Calibrated compound(s) not found

=====================================================================
```

สำเนาไม่ควบคุม
บริษัท ห้องปฏิบัติการกลาง (ประเทศไทย) จำกัด
สาขาเชียงใหม่
เอกสารฉบับนี้ ขอสงวนสิทธิ์ให้กับลูกค้าบริษัทฯ เท่านั้น

=====================================================================

Area Percent Report

=====================================================================

Sorted By : Signal
Calib. Data Modified : 4/6/2020 15:26:21 PM
Multiplier : 1.0000
Dilution : 1.0000
Use Multiplier & Dilution Factor with ISTDs

Signal 1: DAD1 A, Sig=244,8 Ref=off

| Peak # | RetTime [min] | Type | Width [min] | Area [mAU*s] | Area % | Name |
|---|---|---|---|---|---|---|
| 1 | 3.143 | | 0.0000 | 0.00000 | 0.0000 | Vitamin C |

Totals : 0.00000

Results obtained with enhanced integrator!
1 Warnings or Errors :

Warning : Calibrated compound(s) not found

=====================================================================

*** End of Report ***

สำเนาไม่ควบคุม
บริษัท ห้องปฏิบัติการกลาง (ประเทศไทย) จำกัด
สาขาเชียงใหม่
เอกสารฉบับนี้ ขอสงวนสิทธิ์ให้กับลูกค้าบริษัทฯ เท่านั้น

=====================================================================

Injection Date : 2/6/2020 18:36:23 PM Seq. Line : 4
Sample Name : std 0.005 Location : Vial 3
Acq. Operator : supansa Inj : 1
Acq. Instrument : Instrument 1 Inj Volume : 20 µl
Acq. Method : C:\HPCHEM\1\METHODS\VIT_C.M
Last changed : 18/11/2019 15:08:57 PM by supansa
Analysis Method : C:\HPCHEM\1\METHODS\VC060263.M
Last changed : 4/6/2020 15:26:22 PM by supansa

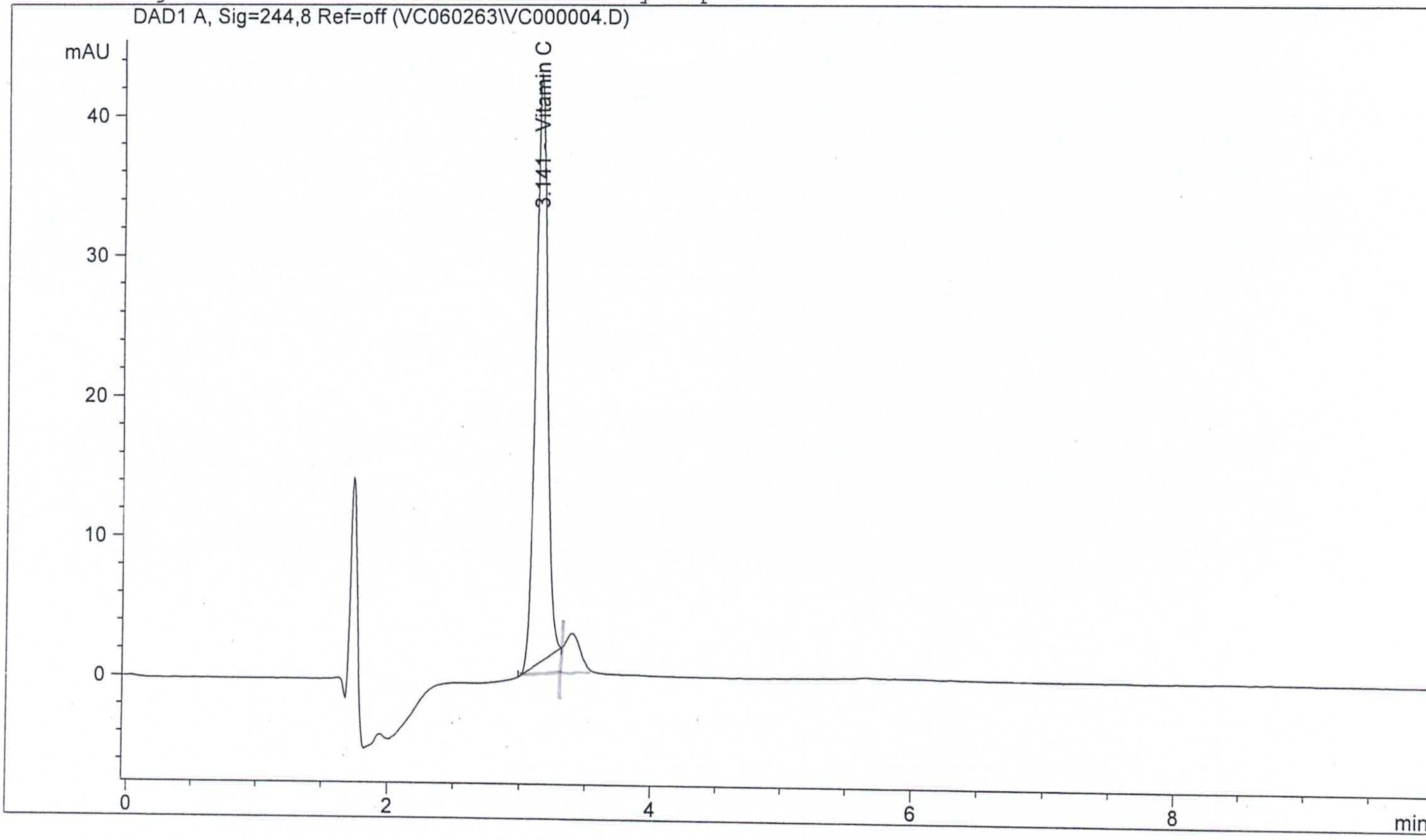


=====================================================================

External Standard Report

=====================================================================

Sorted By : Signal
Calib. Data Modified : 4/6/2020 15:26:21 PM
Multiplier : 1.0000
Dilution : 1.0000
Use Multiplier & Dilution Factor with ISTDs

Signal 1: DAD1 A, Sig=244,8 Ref=off

| RetTime [min] | Type | Area [mAU*s] | Amt/Area | Amount [g/L] | Grp | Name |
|---|---|---|---|---|---|---|
| 3.141 | BP | 254.34193 | 1.91700e-5 | 4.87574e-3 | | Vitamin C |

Totals : 4.87574e-3

Results obtained with enhanced integrator!

=====================================================================

*** End of Report ***

สำเนาไม่ควบคุม
บริษัท ห้องปฏิบัติการกลาง (ประเทศไทย) จำกัด
สาขาเชียงใหม่
เอกสารฉบับนี้ ขอสงวนสิทธิ์ให้กับลูกค้าบริษัทฯ เท่านั้น

```
=====================================================================
Injection Date  : 2/6/2020 18:48:34 PM              Seq. Line :   5
Sample Name     : std 0.01                           Location : Vial 4
Acq. Operator   : supansa                                 Inj :   1
Acq. Instrument : Instrument 1                     Inj Volume : 20 µl
Acq. Method     : C:\HPCHEM\1\METHODS\VIT_C.M
Last changed    : 18/11/2019 15:08:57 PM by supansa
Analysis Method : C:\HPCHEM\1\METHODS\VC060263.M
Last changed    : 4/6/2020 15:26:22 PM by supansa
```

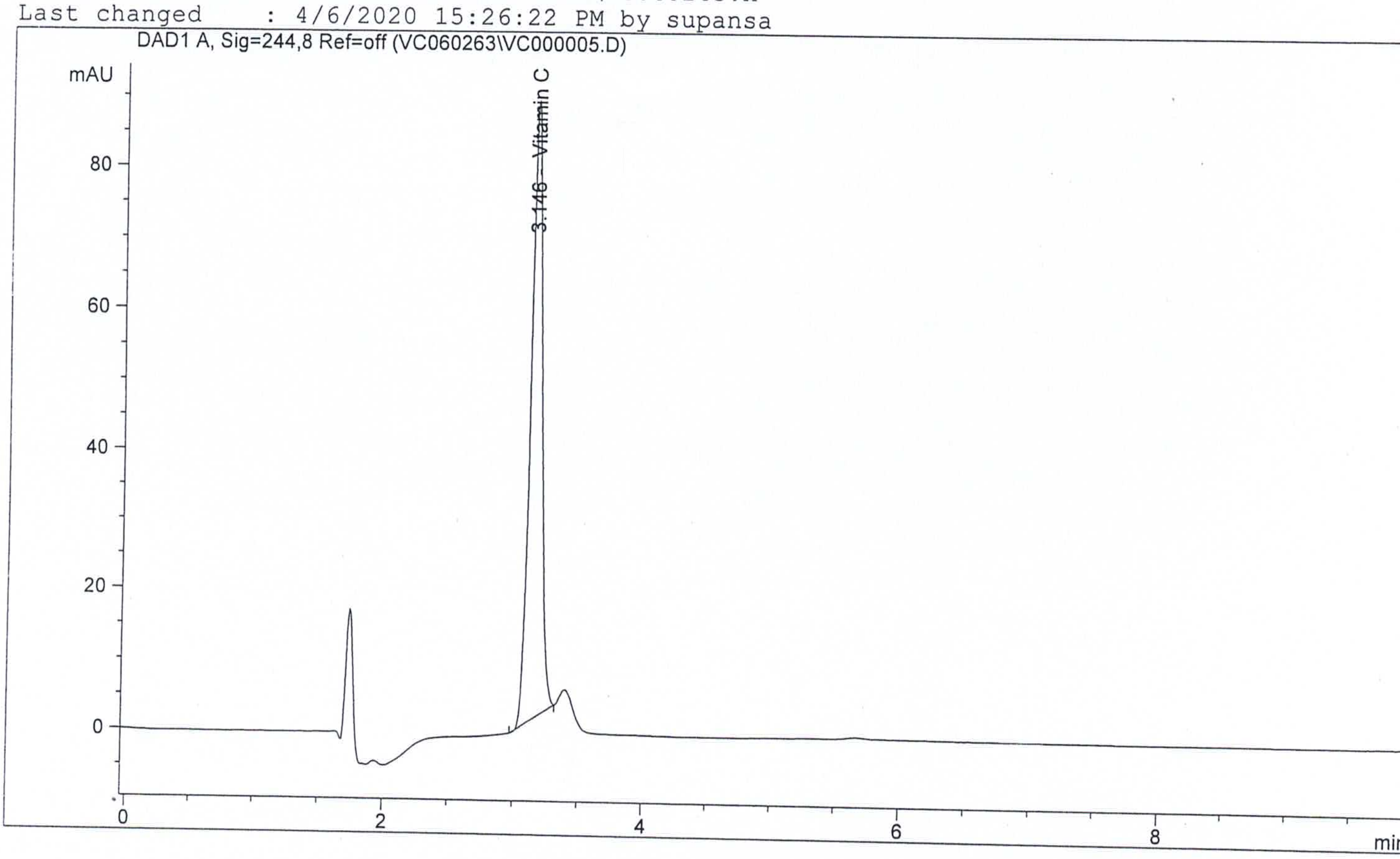


```
=====================================================================
                     External Standard Report
=====================================================================

Sorted By             :      Signal
Calib. Data Modified  :      4/6/2020 15:26:21 PM
Multiplier            :      1.0000
Dilution              :      1.0000
Use Multiplier & Dilution Factor with ISTDs

Signal 1: DAD1 A, Sig=244,8 Ref=off

RetTime  Type    Area       Amt/Area     Amount     Grp   Name
 [min]         [mAU*s]                   [g/L]
-------|------|----------|----------|----------|--|------------------
  3.146 BP     531.21008 1.84648e-5 9.80871e-3     Vitamin C

Totals :                             9.80871e-3

 Results obtained with enhanced integrator!
=====================================================================
                        *** End of Report ***
```

สำเนาไม่ควบคุม
บริษัท ห้องปฏิบัติการกลาง (ประเทศไทย) จำกัด
สาขาเชียงใหม่
(เอกสารฉบับนี้ ขอสงวนสิทธิ์ให้กับลูกค้าบริษัทฯ เท่านั้น)

```
=====================================================================
Injection Date  : 2/6/2020 20:26:31 PM         Seq. Line :   13
Sample Name     : 04926-001                      Location : Vial 12
Acq. Operator   : supansa                             Inj :   1
Acq. Instrument : Instrument 1                 Inj Volume : 20 µl
Acq. Method     : C:\HPCHEM\1\METHODS\VIT_C.M
Last changed    : 18/11/2019 15:08:57 PM by supansa
Analysis Method : C:\HPCHEM\1\METHODS\VC060263.M
Last changed    : 4/6/2020 15:26:22 PM by supansa
```

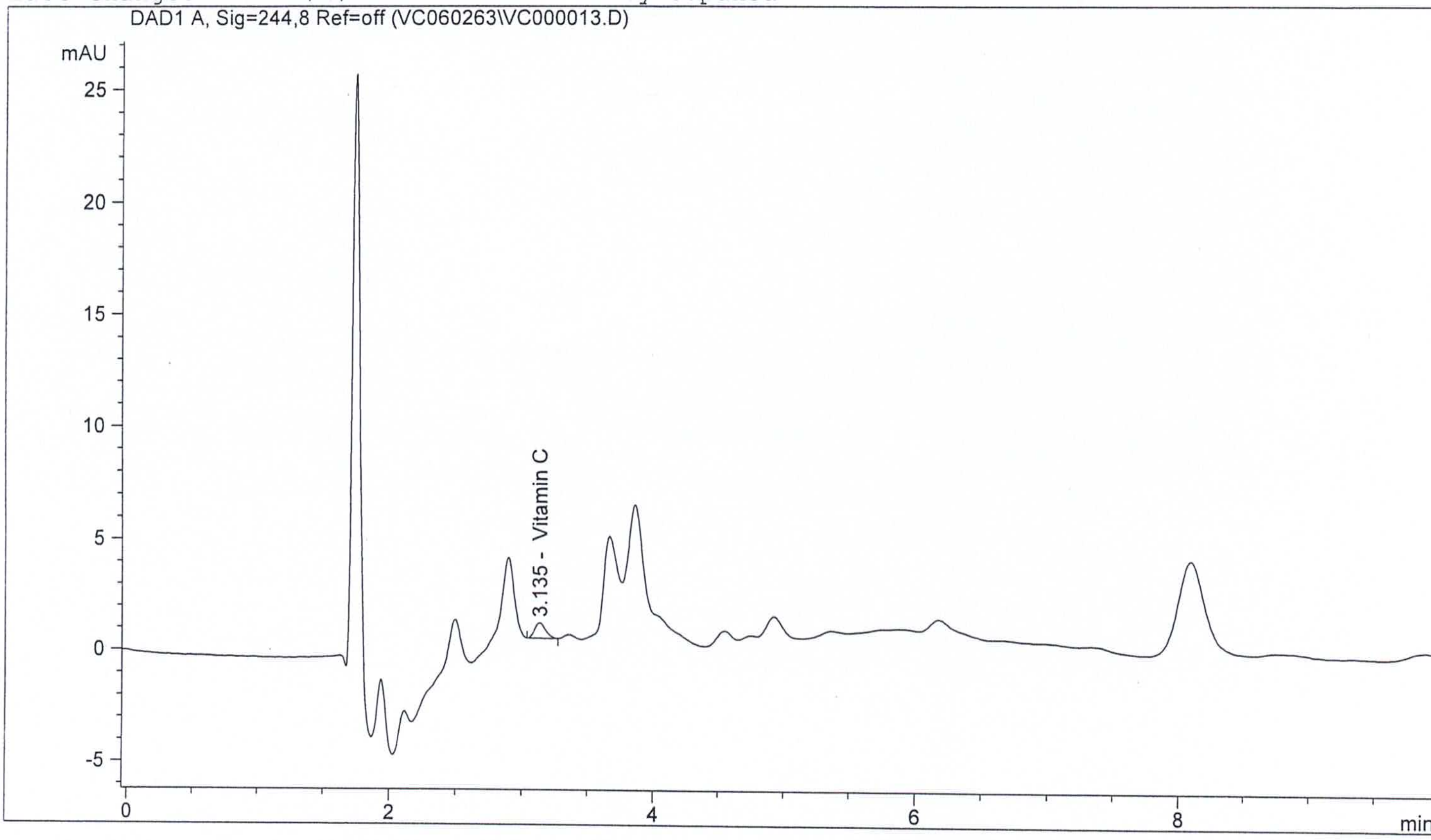


```
=====================================================================
                      External Standard Report
=====================================================================

Sorted By             :      Signal
Calib. Data Modified  :      4/6/2020 15:26:21 PM
Multiplier            :      1.0000
Dilution              :      1.0000
Use Multiplier & Dilution Factor with ISTDs

Signal 1: DAD1 A, Sig=244,8 Ref=off
```

| RetTime [min] | Type | Area [mAU*s] | Amt/Area | Amount [g/L] | Grp | Name |
|---|---|---|---|---|---|---|
| 3.135 | BP | 4.05048 | 1.02774e-4 | 4.16283e-4 | | Vitamin C |

```
Totals :                               4.16283e-4

 Results obtained with enhanced integrator!
=====================================================================
                       *** End of Report ***
```

สำเนาไม่ควบคุม

บริษัท ห้องปฏิบัติการกลาง (ประเทศไทย) จำกัด

สาขาเชียงใหม่

เอกสารฉบับนี้ ขอสงวนสิทธิ์ให้กับลูกค้าบริษัทฯ เท่านั้น

=====================================================================

```
Injection Date      : 2/6/2020 20:38:45 PM          Seq. Line :  14
Sample Name         : 04926-002                      Location : Vial 13
Acq. Operator       : supansa                             Inj :  1
Acq. Instrument     : Instrument 1                 Inj Volume : 20 µl
Acq. Method         : C:\HPCHEM\1\METHODS\VIT_C.M
Last changed        : 18/11/2019 15:08:57 PM by supansa
Analysis Method     : C:\HPCHEM\1\METHODS\VC060263.M
Last changed        : 4/6/2020 15:26:22 PM by supansa
```

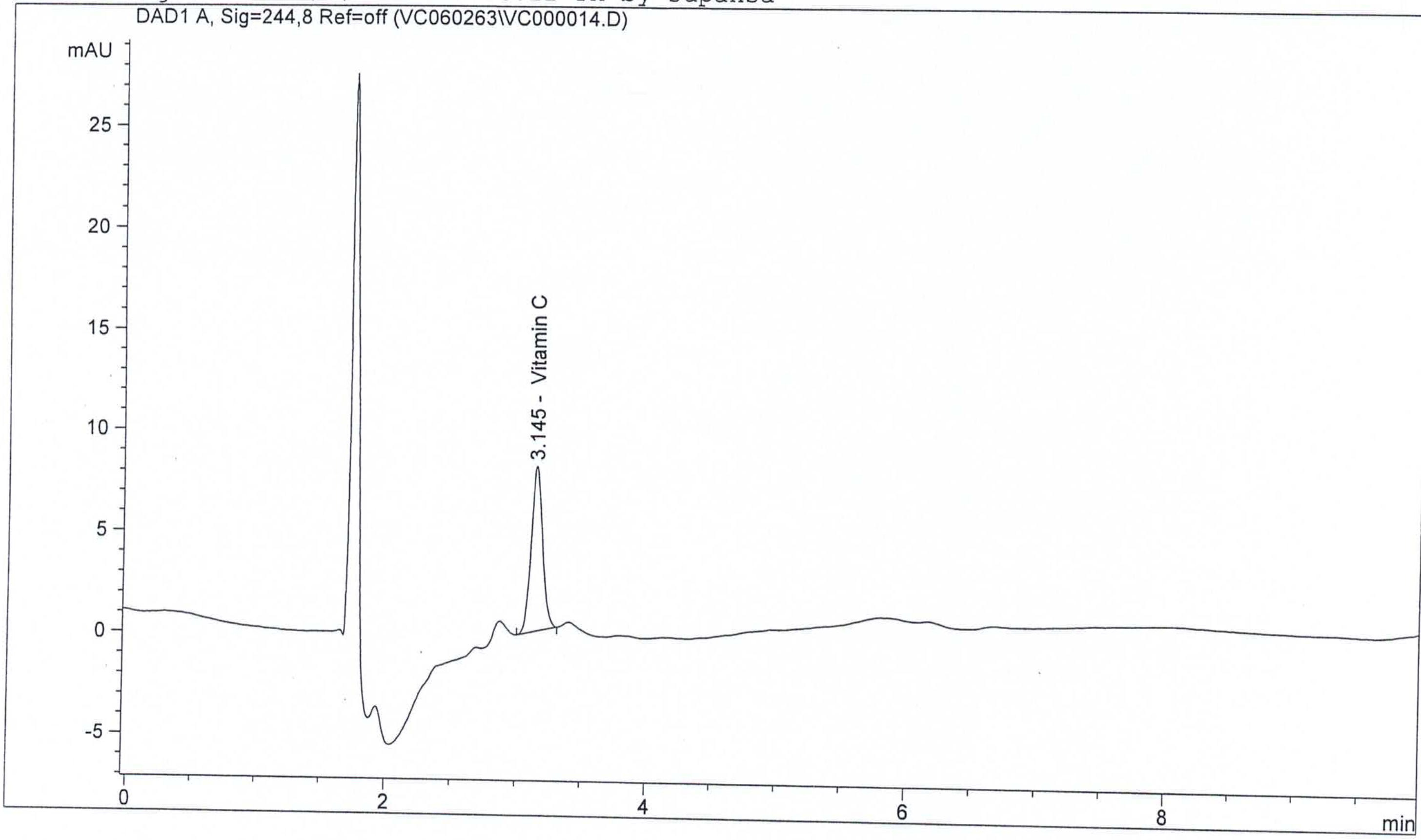


=====================================================================

External Standard Report

=====================================================================

```
Sorted By             :      Signal
Calib. Data Modified  :      4/6/2020 15:26:21 PM
Multiplier            :      1.0000
Dilution              :      1.0000
Use Multiplier & Dilution Factor with ISTDs
```

Signal 1: DAD1 A, Sig=244,8 Ref=off

| RetTime [min] | Type | Area [mAU*s] | Amt/Area | Amount [g/L] | Grp | Name |
|---|---|---|---|---|---|---|
| 3.145 | PP | 49.60273 | 2.47545e-5 | 1.22789e-3 | | Vitamin C |

Totals : 1.22789e-3

Results obtained with enhanced integrator!

=====================================================================

*** End of Report ***

สำเนาไม่ควบคุม
บริษัท ห้องปฏิบัติการกลาง (ประเทศไทย) จำกัด
สาขาเชียงใหม่
เอกสารฉบับนี้ ขอสงวนสิทธิ์ [illegible]
